# Reporting randomised trials of physical exercise or training interventions in older adults: the PETIO guideline

**DOI:** 10.1186/s11556-025-00390-x

**Published:** 2025-12-02

**Authors:** Bettina Wollesen, Piesie A. G. Asuako, Mona Herden, Christoforos D. Giannaki, Antoine Langeard, Nicola Lamberti, Melanie Mack, Michel Audiffren, Yael Netz, Claudia Voelcker-Rehage

**Affiliations:** 1 Institute of Movement Therapy and Movement-Oriented Prevention and Rehabilitation, German Sport University Cologne, Cologne, Germany; 2 Department of Neuromotor Behavior and Exercise, University of Münster, Münster, Germany; 3 Department of Human Movement Science, Universität Hamburg, Hamburg, Germany; 4 Department of Life Sciences, School of Life and Health Sciences, University of Nicosia, Nicosia, Cyprus; 5 INSERM, COMETE U1075, CYCERON, CHU de Caen, Université de Caen Normandie, Caen, France; 6 Department of Neuroscience and Rehabilitation, University of Ferrara, Ferrara, Italy; 7 Center for the Interdisciplinary Study of Gerontology and Vulnerability, University of Geneva, Geneva, Switzerland; 8 Research Center on Cognition and Learning, University of Poitiers, Poitiers, France; 9 Levinsky-Wingate Academic College, Wingate Campus, Israel

**Keywords:** Consensus, Consistency, CONSORT, Delphi, Exercise intensity, Older adults

## Abstract

**Objective:**

This reporting guideline was developed to address the gap in methodological reporting standards for trials investigating physical exercise or training in older adults, aiming to enhance the quality, transparency, and replicability of such research. The aim is to improve the reporting of key elements, including population characteristics, intervention components [e.g., Frequency, Intensity, Time, Type (FITT) principles, tailoring, use of technology], study design and methods (e.g., recruitment, randomization, statistical analysis), as well as study results, including outcomes and adherence measures.

**Methods:**

A six-stage process was used to develop this guideline. This included a three-round Delphi process involving experts from a large European network (COST Action PhysAgeNet), a comprehensive literature review of existing reporting guidelines, consensus meetings with international experts, and validation with journal editors who evaluated and refined the guideline.

**Results:**

The final PETIO guideline includes an expanded checklist of items to report in the context of physical exercise interventions in older adults. Experts and editors agreed on essential items for improving quality, transparency, and replicability, such as intervention components (FITT) and setting, tailoring aspects, adverse events, and control group specifications. Notably, it was also emphasized that standardized reporting is critical for future meta-analyses and the implementation of future research protocols.

**Conclusion:**

The guideline is expected to support researchers, peer reviewers, and journal editors in improving the quality and transparency of research on physical exercise interventions in older adults.

**Release date:**

2025 (original version).

**Availability:**

The guideline is freely accessible online in the supplemental material.

**Supplementary Information:**

The online version contains supplementary material available at 10.1186/s11556-025-00390-x.

## Introduction

The global population is rapidly aging, with the number of people aged 60 and older expected to double by 2050 [1]^1^. To address this demographic shift, effective interventions are needed to promote the health and well-being of older adults. Maintaining health and well-being in later life not only enhances quality of life and independence but also reduces the burden on healthcare systems and caregivers. Exercise interventions have demonstrated a wide range of benefits, including improved mobility and fall prevention [2], enhanced well-being [3], better cognition [4], and a reduced risk of chronic diseases [5] and depression [6].

Comprehensive reporting of exercise interventions in scientific research is crucial for the reproducibility of findings, facilitating systematic literature reviews and meta-analyses, and supporting the development of evidence-based, tailored exercise programmes for older adults [7]. Importantly, complete reporting of exercise interventions increases transparency, enabling readers to evaluate the methodological quality of research and, consequently, to understand the extent to which the results may be biased [8]. Furthermore, it allows researchers to systematically address deficiencies in the evidence base.

The EQUATOR Network (Enhancing the Quality and Transparency of Health Research) has advocated the use of discipline-specific reporting guidelines - including those in the fields of rehabilitation and aging - to improve the accuracy, transparency, and reliability of health research [9, 10]. Inconsistent and incomplete reporting poses a significant barrier to the replication of studies, the synthesis of findings through systematic reviews, and the effective translation of research into practice.

Physical exercise interventions face particular challenges due to their complexity, multimodal nature, and the necessity for personalized prescription. A review of systematic reviews examining the reporting quality of physical exercise interventions across various health conditions found that, compared to drug trials, exercise trials often demonstrate lower methodological quality, a higher risk of bias, and less frequent reporting of adverse events [11, 12]. Consistent with these findings, several recent studies have evaluated the quality of reporting tools used for physical exercise interventions. For instance, Kattackal et al. [13] compared three commonly used checklists - TIDieR (Template for Intervention Description and Replication), CERT (Consensus on Exercise Reporting Template), and i-CONTENT (Consensus on Therapeutic Exercise Training; [14]) - in the context of juvenile idiopathic arthritis. Their results suggest that the inclusion of supplemental information on physical exercise programs may improve the quality of reporting in randomized controlled trials (RCTs) and facilitate the reproducibility of interventions in both research and clinical practice. In a systematic review of exercise interventions for low back pain, Davidson et al. [15] found that reporting quality (as evaluated using the CERT and TIDIeR checklists) was often poor, with major gaps in essential components such as tailoring or exercise dosage. This may be because the current reporting guidelines lack specificity regarding exercise-based interventions and the contexts in which they are used. The authors concluded that further work is required to improve reporting [15], by providing more precise guidelines and assisting authors in adhering to them.

Although these studies demonstrate that reporting tools help to standardize the reporting of exercise interventions (for example cf. [16, 17]), they lack important specificities relevant to studies involving exercise in older adults. For instance, while they may require dose details to be reported, they often lack a specific, standardized framework -such as the FITT principles - to ensure this information is reported consistently and comprehensively. In older populations, researchers are confronted with high heterogeneity, multimorbidity, and diverse psychosocial aspects [1, 18, 19], which usually should not affect the reporting of the results but increase the number of variables to consider, thereby raising the risk of overlooking important aspects when describing the results. There is accumulating evidence that these diverse contextual factors modify intervention effects in older adults. For example, multimorbidity profiles have been found to alter the magnitude of exercise benefits [20], while baseline physical and functional status (e.g., gait speed) has been found to correlate with the size and direction of intervention effects [21]. Cognitive status (e.g. MCI, dementia) is also repeatedly found to impact adherence and intervention effects, indicating that adherence requirements and delivery need to differ from those for cognitively intact older adults [22, 23]. Mental health similarly influences intervention effects, as baseline depression symptoms are associated with poorer adherence, thereby attenuating benefits [24, 25]. Moreover, access to technology, digital literacy, and the availability of usability are critical factors the influence engagement in in technology-supported exercises and should be considered when interpreting study findings [26, 27].

This necessitates the careful selection, documentation, and reporting of study designs and methodological features [28–30]. The health, physical, mental, and functional characteristics of older participants, as well as their digital literacy and access to technology as additional contextual factors, need to be reported more thoroughly.

Detailed information on the intervention, which can also be specific to older adults, is often lacking. This includes information on the frequency, intensity, time, and type of exercise (FITT) [31, 32], as well as the setting in which the intervention took place (e.g., nursing homes, community centers, or home-based settings) and the level of adherence or reasons for dropout [33, 34]. Although some guidelines recommend reporting these aspects, many scientific papers omit such details, making it difficult to understand the factors contributing to an intervention’s success or to replicate it across different older populations [35]. This also limits the inclusion and utility of studies in meta-analyses, constraining risk of bias assessment and the ability to conduct analyses exploring underlying mechanisms, such as moderation or mediation analyses. Ultimately, this hinders scalability and implementation [17, 36].

Further emphasizing the need for methodological rigor, a lack of reporting was observed regarding the connection between the research hypothesis and the outcomes of interest, as well as the inclusion of theoretical models underlying the proposed intervention [37].

In summary, the existing reporting guidelines [16, 38], which aim to improve the transparency of exercise and rehabilitation interventions, are not tailored for use in trials involving older adults. As a result, key descriptors of exercise interventions may be incompletely reported in this population [35]. Some guidelines were specifically designed for clinical settings [38], rather than for physical training in older adults, and are therefore too general with respect to principles such as F.I.T.T. In addition, none of the existing frameworks address digital delivery, despite its growing relevance for feasibility and adherence among older populations. These limitations underscore the need for PETIO as a population - and context-specific extension that complements existing tools.

While exercise programs for older adults frequently rely on non-randomized or single-arm intervention designs due to feasibility and ethical considerations, our focus on randomized controlled trials was deliberate. Randomized controlled trials remain the gold standard for assessing the effectiveness of interventions, as they minimize bias and enable stronger causal inference. In developing reporting guidelines, prioritizing randomized interventions ensures that the foundation rests on studies with the highest methodological rigor.

To sum up, although reporting guidelines for RCTs already exist and the effects of physical exercise on various health outcomes in older adults are well documented, the absence of standardized reporting of exercise interventions (e.g., including FITT principles and other training characteristics) in older adults not only limits the generalizability of findings but also hampers the development of tailored, evidence-based physical exercise programs that meet the needs of diverse ageing populations. Comprehensive and consistent reporting would enable researchers and practitioners to identify evidence-based exercise recommendations, replicate studies, and ultimately establish more robust frameworks for physical activity in older adults.

**Fig. 1:**
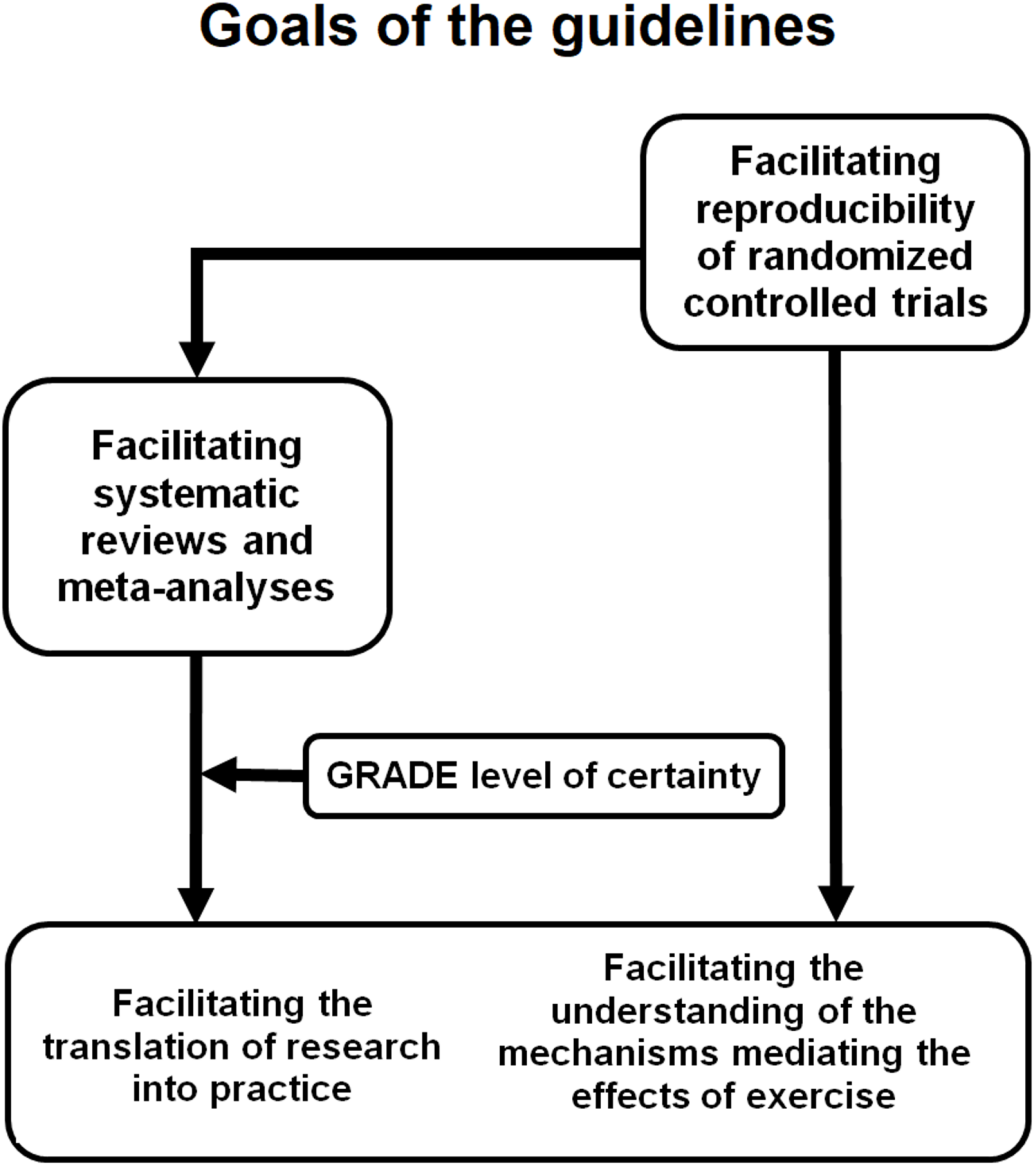
Objectives of the PETIO reporting guideline for exercise in older adults

Building on the existing Consolidated Standards of Reporting Trials (CONSORT) guidelines for randomized controlled trials and taking into account the specific characteristics of exercise interventions in older adults, we present an extended reporting guideline designed to improve the transparency, quality, and completeness of exercise intervention descriptions, and to ensure that the demonstrated benefits for older adults can be accurately interpreted, replicated, and translated into practice (see Fig. 1 for an overview of the guidelines’ objectives).

## Methodology

**Fig. 2:**
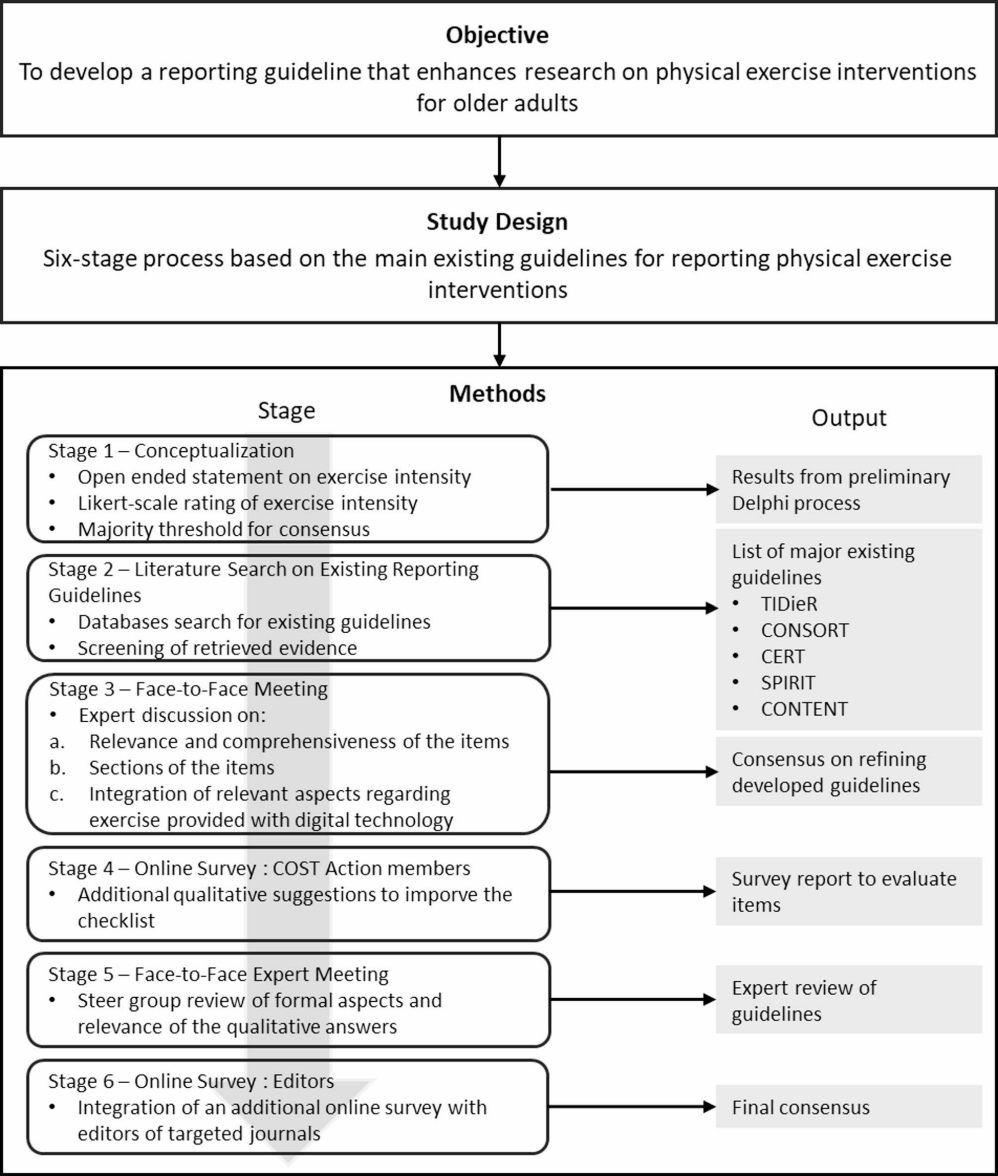
Overview of the development process of the PETIO guideline

Figure 2 illustrates the six-stage process undertaken in the development of the guideline for this project. This involved a rigorous and systematic approach, incorporating a comprehensive review of existing reporting standards, consultations with experts in geriatrics and exercise science, and iterative refinement based on stakeholder feedback, guided by the principles outlined by Moher [39]. After forming an interdisciplinary development group with experts from the field (e.g., exercise and human movement scientists, exercise physiologists, health professionals, and psychologists), the process comprised the results of an initial Delphi survey (Stage 1), literature reviews (Stage 2), face-to-face meetings (Stages 3 and 5), group discussions (Stages 3 and 5), and consensus questionnaires (Stages 4 and 6). The main focus was on reporting randomized controlled trials (RCTs) examining the effects of physical exercise interventions in older adults. Overall, the development process was conducted in six stages (cf. Fig. 2):

### Stage 1 – conceptualization: preliminary Delphi process

The guideline development process was based on a modified Delphi study comprising three rounds, which provided expert recommendations for categorizing exercise intensity in older adults [7]. All three Delphi rounds were sent via email to the members (*N* = 297) of the EU COST Action “PhysAgeNet”. Participation in the process required informed consent (see [7] for further information).

Round one of the Delphi process included four blocks of questions, involving the collection of open-ended statements from the PhysAgeNet consortium that defined light, moderate, vigorous, and high intensities, as well as recommendations to improve the reporting quality of exercise interventions. This round received 93 valid responses. In round two, these statements retrieved from the first round were rated for agreement using the Likert-scale rating (ranging from 0 = totally disagree to 10 = totally agree), with 67 responses received. The third round applied a simple majority threshold to determine overall agreement on the recommendations for reporting exercise intensity when no objective or subjective measurement was available, receiving responses from 72 experts.

### Stage 2 – literature search for existing reporting guidelines

Secondly, a comprehensive literature search was conducted using the following electronic databases: PubMed, Google Scholar, Scopus, and Web of Science, to identify literature on existing guidelines for reporting physical exercise. No restrictions were applied regarding the date of publication. The following criteria were applied to retrieve relevant existing guidelines from the databases:

- Study type: Reporting guidelines specifically developed for randomized controlled trials (RCTs), such as CONSORT [40];
- Intervention focus: Guidelines that explicitly capture exercise-specific variables, such as FITT, like the CERT [16];
- Reproducibility: Guidelines that provide sufficient detail to enable replication of the intervention;
- Target population: Guidelines applicable to, or designed for use in, studies involving human populations, including older adults;
- Outcome reporting: Guidelines that facilitate clear and comprehensive reporting of study outcomes.

### Stage 3 – face-to-face meeting to develop and refine items

Thirdly, based on the results of the literature search, the CONSORT checklist [40] was selected for further refinement. It was extended to integrate the relevant items identified in Stages 1 and 2. The meeting discussions were based on a preliminary list of items compiled from the results of the Delphi process (Stage 1), the gaps identified in existing reporting guidelines through the literature review (Stage 2), and additional points raised by the core working group based on their expertise in physical exercise training studies in older adults.

The rationale for the introduction took into account key aspects, such as the alignment between the initial hypothesis and the outcome of interest, as well as the underlying theoretical models that informed the design of the interventions. Additionally, participant characteristics were included, such as age range, level of education, physical activity status, and familiarity with technology if the intervention involved technological components. The description of the intervention was structured according to the FITT principles, particularly with regard to exercise intensity and adherence assessment. Finally, aspects related to potential harms and participant dropouts were included, particularly when technology was involved.

Furthermore, the PETIO guideline includes items on the exercise setting and delivery method, as well as detailed descriptions of the materials and equipment used. It also emphasizes the inclusion of motivation strategies designed to support participant engagement and adherence, recognizing their importance in promoting sustained participation and optimizing outcomes.

The extended version of the checklist was presented during a hybrid-format group discussion with experts from the PhysAgeNet COST Action (*N* = 28). This stage of the discussion included three aspects:

1. Relevance and comprehensiveness of the integrated items,
2. CONSORT section in which the new items should be included,
3. Integration of relevant aspects regarding exercise interventions delivered using digital technology.

Moreover, the wording of the items was discussed and, where necessary, revised to ensure clarity and comprehension.

The result was a set of key reporting items covering essential aspects of exercise interventions, such as participant characteristics, intervention components, measures of adherence and compliance, and outcomes.

### Stage 4 – online survey: COST action members (Consensus round 1)

The extended and revised checklist was transferred into an online questionnaire (LimeSurvey.net). The survey incorporated all 108 items of the new checklist and included a rating of the relevance of these items on a 10-point Likert scale (ranging from 0 = totally disagree to 10 = totally agree), as well as the opportunity to provide additional free-text feedback on how to improve the checklist. A link to the survey was sent to all PhysAgeNet members (*N* = 442) via a mailing list. The invitation included an explanation of the process, as well as the timeframe for participation (two weeks, from 14th to 30th of August 2024). All participants were required to provide informed consent before proceeding with the online questionnaire.

### Stage 5 – face-to-face expert meeting: discussion of the PETIO guideline content

The results of the online survey were presented and discussed at an additional face-to-face meeting with members of Working Group 1 of the PhysAgeNet Cost Action. This meeting focused on refining the checklist based on both the quantitative and qualitative feedback. Formal aspects, such as language clarity, grammar, and redundancies in the items, were carefully reviewed. Moreover, the relevance of the qualitative feedback was discussed, and changes to the checklist were implemented following consensus.

### Stage 6 – online survey: editors (Consensus round 2)

The final consensus round consisted of an additional online survey conducted with editors of journals that commonly publish studies on exercise in older adults. The survey asked editors to rate the relevance of the items (*N* = 208) using a 10-point Likert scale (ranging from 0 = totally disagree to 10 = totally agree) and provided space for additional qualitative feedback. Owing to the length of the questionnaire, items from the original CONSORT checklist that were not modified in the final version were excluded.

A total of 311 editors were identified through extensive online research and contacted by email. The email introduced the purpose of the guideline and explained the entire process. Nineteen editors replied to the email invitation: six confirmed their participation, while thirteen declined, citing reasons such as lack of relevant expertise (*n* = 9), unavailability (*n* = 2), and no longer serving as editor-in-chief (*n* = 1). All participating editors provided informed consent at the start of the online questionnaire.

One-way ANOVAs were conducted in SPSS Statistics to analyze potential differences between expert and editor opinions (IBM SPSS Statistics, Armonk, NY, USA Version 29.0.2.0). A two-tailed alpha level of 0.05 was used to determine statistical significance.

## Results

### Results of stage 1 – Delphi process

After sending an initial invitation to members of the COST Action PhysAgeNet, 93 participants replied in the first round, 67 in the second, and 72 in the third round of the Delphi process [7]. The overall agreement score ranged from 6.7 to 8.8 out of 10 points, underscoring the importance of standardized reporting. The outcome of this process was a catalogue of consensus-based dimensions rated as highly important for reporting. Experts recommended providing comprehensive details on the exercise intervention based on the FITT principle, as well as additional information on how the training was controlled, in order to improve the quality of reporting in future intervention studies with older adults. Moreover, experts noted that unreported or uncontrolled intensity often hinders the process of systematic reviews and meta-analyses. The findings of the Delphi process highlighted the need for thorough documentation of exercise interventions to enhance the comparability and validity of such studies. These consensus-derived reporting aspects and their agreement ratings are summarized in Table 1, which presents the most highly rated items identified as crucial for improving the reporting quality of exercise intervention studies with older adults. [7].

**Table 1:** Ratings of relevant reportings to improve reporting quality of exercise studies with older adults

Reporting on physical exercise intervention studies with older adults Describe:	Rating (M ± SD)
Exercise program modality/FITT principle (type, frequency, intensity, duration) (97%)	9.1 ± 1.1
Exercise progression (94%)	9.1 ± 1.2
Training volume (sets, repetitions, minutes) (94%)	9.1 ± 1.3
Type of control group (94%)	9.0 ± 1.6
% of maximum HR max/HR reserve (97%)	8.9 ± 1.1
Physical fitness of the target group at baseline (97%)	8.8 ± 1.6
Exercise Intensity (weight, speed, distance) (92%)	8.7 ± 1.4
Resting Time (92%)	8.7 ± 1.5
Methods used to assess exercise intensity (89%)	8.7 ± 1.5
RPE/Fatigue before and after (94%)	8.6 ± 1.5
Intensity changes across different exercise types (85%)	8.5 ± 1.8
Adverse events such as injuries (89%)	8.3 ± 1.9
Setting (home-based/group) (92%)	8.2 ± 2.0
METs (89%)	8.1 ± 2.5
% of maximal oxygen consumption/intensity at blood lactate threshold (83%)	8.0 ± 2.2
Time of day (75%)	7.5 ± 2.4

*Abbreviations: HR *Heart rate,* RP *Rating of perceived exertion,* METs *Metabolic equivalent of task

### Results from stage 2 – literature search on existing reporting guidelines

The following reporting guidelines met all eligibility criteria and were selected to inform the development of the current guidelines: CERT [16, 41], CONSORT [40], i-CONTENT [14], SPIRIT [42], and TIDieR [17]. As these guidelines are applicable to exercise intervention studies, they provided a robust foundation for developing the current guideline. Owing to their relevance to exercise intervention research, CERT, CONSORT, SPIRIT, and TIDieR guidelines were further evaluated to integrate pertinent aspects of exercise studies, including those involving older adults.

The CERT checklist was specifically developed for reporting exercise interventions and includes detailed information on, for example, equipment, instruction, and delivery format [16]. The CERT consists of 19 items grouped into seven categories: (1) materials used, (2) provider characteristics, (3) mode of delivery, (4) setting, (5) dosage (frequency, intensity, time, and type), (6) tailoring, and (7) adherence. Despite its strengths, the guideline lacks specific recommendations for designing and reporting exercise intervention studies involving older adults (e.g., fitness level and health conditions), particularly with regard to the heterogeneity of aging in terms of physical, mental, and psychosocial characteristics, as well as the new modes of delivery emerging from technological advancements.

The CONSORT statement provides a robust framework for transparent and standardized reporting of randomized controlled trials [40]. It comprises a flow diagram and a 25-item checklist (37 elements in total) designed to enhance the completeness and reproducibility of RCT reporting. The updated checklist includes 30 items and adds specific information about Open Science after the abstract (five additional aspects previously listed under “Other information”; [43]. While CONSORT is widely adopted and well established for general RCT reporting, it offers limited detail [44], particularly regarding the specifics of exercise interventions designed to meet the needs of older adults, such as multimorbidity or psychosocial characteristics, which are especially relevant in this populations.

The i-CONTENT scale [14] was developed for the implementation of therapeutic exercise programs; however, limited information is available regarding its development process. It comprises five specific item categories: patient eligibility; provider competences and exercise program setting; rationale; content; and adherence to the exercise program. However, it provides less detail concerning the specific physical and mental characteristics associated with older age. Ultimately, the i-CONTENT checklist was excluded as a foundational source owing to the limited information available on its development process, despite its relevance to the implementation of therapeutic exercise programs.

The SPIRIT checklist was developed to improve the quality of reporting in RCT protocols [42]. It outlines 33 items that should be addressed when preparing trial protocols, covering areas such as trial design, participant eligibility, interventions, and outcome measures. SPIRIT provides guidance to ensure methodological transparency. In the updated checklist, the integration of patient and public involvement has been added, bringing the total to 34 items [45]. However, similar to the CONSORT guidelines, it does not provide tailored guidance for reporting complex exercise-based intervention RCTs involving older adults, particularly in relation to areas such as digital literacy, comorbidities, and psychosocial variability.

The TIDieR checklist was developed as an extension of the CONSORT and SPIRIT guidelines to enhance the reporting of interventions, including exercise interventions [17]. Although it provides a solid foundation for reporting, it does not capture all relevant details of exercise interventions, such as dosage or how interventions are tailored [13, 15].

Additionally, there is the TIDieR-Rehab checklist, developed as an extension of TIDieR specifically for rehabilitation interventions. This checklist includes additional items relevant to interventional research in older adults but lacks details on training, control and management, and the use of assistive technology.

Taken together, key domains from CERT, CONSORT, SPIRIT, and TIDieR were integrated into the current guideline. These include materials used in the intervention; provider characteristics (e.g., whether the intervention was supervised); mode and location of delivery; timing and dosage; intervention tailoring; and participant adherence. Additional elements adopted from the four main guidelines include identifying the study as an RCT in the title, detailed reporting of participant flow, and structured summaries of the trial design, methods, results, and primary and secondary outcomes, as well as the conclusions.

### Results of stage 3 – face-to-face meeting: item development and refinement

After reviewing the results of the Delphi process and the literature on existing reporting guidelines, the expert group decided to extend the CONSORT guidelines, as they incorporate the largest number of relevant items to be reported. However, extensions were suggested for some sections.

The expert group recommended that the abstract include detailed information on participants, including age and sex distribution, and basic characteristics of the intervention, including type, frequency, duration, and intensity. The abstract is often the first - and sometimes the only - part read in full (and screened within a systematic review). Without these critical details, readers cannot accurately assess the study’s relevance, applicability, or potential impact. Including age and sex in the abstract allows readers to quickly determine whether the study population matches their context or target demographic. Furthermore, the FITT principle (Frequency, Intensity, Time, and Type) provides a standardized framework for describing exercise interventions. These components directly influence the physiological and psychological outcomes in older adults and, in turn, determine the specific health benefits achieved [31]. Clearly reporting the FITT elements in the abstract facilitates a rapid assessment of the intervention’s structure, feasibility, and replicability.

In the introduction, a new key addition was made: the specification of the theoretical framework in the study rationale. The rationale sets the stage by explaining why the study is necessary and which gaps it aims to address. Exercise interventions for older adults are often designed to improve specific physical, mental, and social health outcomes, yet many studies fail to clearly articulate why particular interventions are chosen or needed. This omission can impede understanding of the study’s objectives and relevance, and compromise research transparency.

Thirdly, in the methods section, the expert group argued that, while abstracts may briefly summarize the population and key intervention characteristics to enable rapid categorization of a study, a comprehensive description of diverse population subgroups (e.g., varying health statuses, fitness levels, and cognitive abilities) and intervention specifics is essential to ensure accurate interpretation and reproducibility. The expert group recommended including detailed information on participant and intervention characteristics for both the experimental and control groups. They also recommended reporting compliance assessments and potential confounding factors, such as additional non-exercise components in the intervention group.

Regarding the reporting of participant characteristics, they noted that a high level of detail is particularly vital for geriatric populations, where heterogeneity in health status, medication use, and functional capacity can significantly influence intervention outcomes.

The reporting of participant characteristics could include (but not be limited to) baseline assessments of health status (comorbidities), education level, cognition, depressive state, or medication, as these factors are all known to interact with exercise training-related benefits in older populations [46–49].

Regarding the reporting of the intervention itself, the expert group noted that not only the FITT principle but also additional intervention characteristics should be reported in detail, as they are known to moderate the effects of exercise interventions. For example, in technology-assisted interventions, the use of tools such as wearable activity trackers or virtual reality exergames should be clearly described and reported, as these can enhance engagement by providing real-time feedback and incorporating gamification elements [50]. The expert group considered the reporting of technological aspects important, as these can also influence motivational strategies, such as goal setting and social support (e.g., via online platforms), and improve adherence by addressing both intrinsic and extrinsic motivators [51, 52].

Compliance with exercise protocols is another critical moderator of intervention success that should be reported precisely, as older adults with higher compliance rates, for example, tend to show greater improvements in cognitive function compared with those with irregular or sporadic participation [47]. Reporting compliance metrics, such as attendance records or data from wearable devices, is considered essential for distinguishing between the efficacy of the intervention itself and potential failures in its implementation.

The expert group also emphasizes the importance of the nature of the control group, as this represents another key factor moderating intervention effects. When using passive control conditions (e.g., waitlist or usual care), observed benefits in the intervention group may appear inflated, partly due to unaccounted social interaction effects. This is particularly important when reporting studies involving older adults, as social engagement plays a significant role in shaping a range of health-related outcomes [53, 54]. Additionally, non-exercise components, such as nutritional counselling or cognitive training, can further confound the effects of interventions and must be reported in detail. Studies, for example, indicate that incorporating non-exercise components into physical interventions can influence the interpretation of outcomes across multiple domains [55]. However, it is not only the inclusion of such components but also the manner in which they are integrated that should be reported. For example, regarding physical outcomes, both simultaneous and sequential physical–cognitive training demonstrated comparable efficacy to exercise alone and significantly outperformed other control conditions. Exergaming, however, ranked low for both physical and cognitive outcomes [56].

In the final step, the resulting revised checklist was edited for language. The group defined unfamiliar terms and clarified items that might lead to misinterpretation (*N* = 12), resulting in 5 additional items through reorganization.

The resulting version (cf. Supplemental Material 1) was prepared for expert rating in stage 5.

### Results of stage 4 – online survey of COST action members (Consensus round 1)

In this stage, the extended and revised checklist was evaluated through an online questionnaire. The ratings of agreement for the items can be found in Table 2. Overall agreement ranged from 7.55 (item 5i2: If applicable, report participants’ beliefs and stereotypes concerning the effects of regular exercise on health) to 10.00 points (item 12a: Describe statistical methods used to compare groups for primary and secondary outcomes) across the different sections. Moreover, a total of 51 free-text statements were provided (see Supplemental Material 2). These statements addressed various aspects, including abstract reporting (*n* = 4), the redundancy, e.g., related to the existing CONSORT guidelines or previous items of the checklist (*n* = 14); the level of detail in item descriptions (*n* = 12); suggestions for additional items (*n* = 2); problems in understanding the items or suggestions for language optimization (*n* = 9); and other aspects (*n* = 10).

**Table 2:** Ratings by expert and editors on the integrated items of PETIO

Items	Expert Ratings (M ± SD)	Editor Ratings (M ± SD)
Title and abstract		
1a) Identify the study as an RCT in the title; if possible, follow the PICO scheme	8.64 ± 1.8	Omitted—identical to CONSORT.
1b) Include a structured summary of trial design, methods, results, and conclusions (for specific guidance, see CONSORT for abstract)	8.96 ± 1.6	9.16 ± 1.4
1b1) Describe the population (healthy, diseased, etc.)	9.54 ± 1.2	9.31 ± 1.2
1b2) If the title cannot incorporate the PICO scheme (Population-Intervention-Comparison-Outcome) due to a word limit, report all PICO criteria in the abstract	8.32 ± 2.1	8.23 ± 2.1
1c) Report age (mean > 60 years; age range)	9.11 ± 1.6	8.94 ± 1.6
1 d) Report the number of males/females	8.36 ± 1.9	9.00 ± 1.7
1e) Report information about the FITT principle	8.07 ± 2.3	7.94 ± 2.4
Introduction		
Background and objectives		
2a) Describe the scientific background and provide the rationale	9.08 ± 1.5	Omitted—identical to CONSORT.
2a1) Describe potential theoretical models or mechanisms (including a summary of previous studies) explaining why the proposed intervention might work and how they relate to the outcome of interest (e.g. cardiovascular adaptations or increases in muscle mass, etc.)	8.96 ± 1.2	9.03 ± 1.2
2b) Include specific objectives or hypotheses	8.92 ± 1.7	9.68 ± 0.6
2b1) Formulate the research question and hypothesis according to the outcomes of interest	9.26 ± 1.0	9.17 ± 1.6
2b2) Justify whether the outcomes align with the research questions	8.22 ± 1.3	8.61 ± 1.8
Methods		
Trial design		
3a) Describe the trial design (such as parallel, cluster, factorial) including the allocation ratio	9.55 ± 0.7	9.55 ± 1.4
3b) Describe any changes to methods after trial commencement (such as eligibility criteria), including the reasons	9.55 ± 0.7	9.32 ± 1.4
Participants		
4a) Report eligibility criteria for participants, including:	10.00 ± 0.0	9.35 ± 1.8
4a1) Mean or median age of the sample, depending on its distribution, and age range	9.50 ± 1.3	
4a2) Inclusion/exclusion criteria	9.50 ± 2.0	9.74 ± 0.8
4b) Provide a table comparing the main characteristics of the different participant groups, including:	8.11 ± 3.0	9.00 ± 2.1
4b1) “PA level at baseline” and at the end of the intervention; change to “PAlevel/functional capacity at baseline”	7.95 ± 2.8	
4b2) Percentage of women in each group	8.90 ± 2.0	8.80 ± 2.1
4b3) Mean level of education (and SD) of participants in each group; specify whether in years or degree, and clarify but leave the choice to the author.	7.85 ± 2.3	6.67 ± 2.7
4b4) Health status (objective and/or subjective?)	9.11 ± 1.5	8.48 ± 2.2
4b5) If the study design includes technology support or usage, include participants’ experience with technology	8.75 ± 2.2	8.41 ± 1.4
4b6) Participants’ access to the technology	8.10 ± 2.5	8.43 ± 1.3
4b7) Digital literacy	8.37 ± 2.4	7.80 ± 1.9
4b8) Aspects of tailoring the technology	7.90 ± 2.8	8.03 ± 2.0
4c) Describe the settings and locations where the data were collected	8.65 ± 2.0	8.87 ± 1.5
Interventions		
5) Describe the interventions for each group with sufficient detail. If multimodal, provide details for every modality to allow replication, including how and when they were actually administered., and include a description of:	9.67 ± 0.7	9.61 ± 0.7
5a1) Exercise type (single mode or multimodal)	9.85 ± 0.4	9.45 ± 1.1
5a2) Exercise frequency (number of sessions per week)	9.90 ± 0.3	9.65 ± 0.7
5a3) Duration of exercise (length of time spent on each exercise session in the intervention (in minutes))	9.80 ± 0.4	9.77 ± 0.5
5a4) Exercise intensity (level of difficulty or effort exerted during the exercise in the intervention, and methods used to assess and monitor it, e.g., percentage of HR max/HR reserve/VO2max/VO2peak; RPE/fatigue before and after intervention; also/report the scale used (i.e., 6–20, 1–10))	9.80 ± 0.4	9.35 ± 1.2
5b) Describe exercise physiology aspects (cardiovascular, metabolic, and muscular adaptations), Including a description of:	8.56 ± 2.3	9.47 ± 0.7
5b1) Exercise progression (increase in difficulty, duration, and frequency of the exercise program)	9.60 ± 0.8	
5b2) Intensity changes in different exercise types	9.00 ± 2.2	9.39 ± 0.9
5b3) Resting times (duration of rest or recovery between sets or exercises during the intervention)	9.40 ± 1.3	9.23 ± 1.0
5b4) METs for the exercise program/its components (referring to the estimated energy expenditure during the physical activity intervention)	8.05 ± 2.3	8.00 ± 1.8
5b5) If technology was used, describe whether and how exposure is controlled within the technology	9.16 ± 1.2	8.57 ± 1.7
5c) Describe whether any non-exercise components are included	8.10 ± 2.4	9.30 ± 1.2
5e) Describe the exercise settings, including:	9.41 ± 0.9	8.97 ± 1.5
5e1) Whether exercise is performed individually or in a group	9.70 ± 0.7	
5e2) Whether exercise is supervised or unsupervised, and how it was monitored	9.65 ± 0.9	9.45 ± 0.8
5e3) Any home program component	9.05 ± 2.1	9.16 ± 1.3
5e4) If technology is used, describe how exercise was monitored (e.g., telemonitoring)	8.90 ± 2.2	9.37 ± 0.9
5e5) Indicate whether the exercises are tailored or generic (one-size-fits-all): If tailored, describe how they were adapted to the individual	9.30 ± 0.9	9.73 ± 0.6
5e6) Indicate whether there are any simultaneous or consecutive exercise/intervention components (provide a brief detailed description)	8.37 ± 2.1	9.52 ± 0.9
5e7) Type of exercise equipment	9.30 ± 1.0	9.10 ± 1.5
5e8) If technology is used, describe the design, specific functions (including a list of examples with descriptions), software, interface, adaptation methods, and algorithms	8.95 ± 1.8	8.57 ± 1.5
5e9) If technology is used, describe how progression is programmed	8.80 ± 1.8	8.70 ± 1.4
5e10) If technology is used, describe how outcomes are calculated, if available	8.90 ± 2.0	9.13 ± 1.3
5e11) If technology is used, describe the specific match between the technology used in the intervention and the outcomes	8.90 ± 1.8	8.67 ± 1.6
5f) Describe the type of control group in detail (e.g., was the control group active or passive? If active, what type of activity did they perform?)	9.58 ± 1.0	9.42 ± 1.1
5f1) Report whether the control group was blinded	8.79 ± 2.4	9.48 ± 1.0
5f2) Describe if and how exposure is controlled for the control group in the Technology	8.74 ± 2.1	9.27 ± 1.2
5 g) Describe motivational control aspects	8.11 ± 2.2	9.16 ± 1.5
5g1) Describe how compliance/adherence to exercise is measured/assessed	9.53 ± 0.7	
5g2) Describe motivational strategies and behavioral change techniques, if used	8.63 ± 2.2	9.13 ± 1.4
5g3) If applicable, describe how participants are compensated for study participation	7.95 ± 2.3	8.61 ± 1.7
5 h) Describe the extent to which the intervention was not delivered as planned, if applicable	8.63 ± 2.1	9.26 ± 1.3
5i) Control for confounding factors	8.74 ± 2.1	7.79 ± 2.0
5i1) If applicable, report participants’ beliefs and stereotypes concerning the effects of regular exercise on health	7.55 ± 2.9	
5i2) Physical activity (PA) practiced by participants outside the intervention. The method used to measure PA level should be mentioned (e.g., actimeter, questionnaire)	8.60 ± 2.2	8.73 ± 1.7
5i3) If applicable, report participants’ preference for the different groups in a specific intervention (particularly when several interventions are implemented)	8.10 ± 2.3	8.07 ± 2.0
Outcomes		
6a) Describe the pre-specified primary and secondary outcome measures, including how and when they were assessed	9.80 ± 0.5	Omitted—identical to CONSORT.
6b) Describe any changes to trial outcomes after the trial commenced, including the reasons	9.60 ± 0.8	
Sample size		
7a) Describe how the sample size was determined	9.55 ± 0.8	
7b) When applicable, explain any interim analyses and stopping guidelines	8.80 ± 1.4	
Randomization: Sequence generation		
8a) Describe the method used to generate the random allocation sequence	9.35 ± 0.8	
8b) Describe the type of randomization and provide details of any restriction (such as blocking and block size)	9.35 ± 1.0	
8c) Describe the use of a minimization process (e.g., randomization according to participants’ baseline physical and cognitive levels)	9.20 ± 1.1	
Allocation concealment mechanism		
9) Describe the mechanism used to implement the random allocation sequence (such as sequentially numbered containers), including any steps taken to conceal the sequence until interventions were assigned	9.00 ± 1.3	
Implementation		
10) Report who generated the random allocation sequence, who enrolled the participants, and who assigned participants to the interventions	8.25 ± 2.5	
Masking		
11a) Describe whether masking or blinding was applied, and if so, who was blinded after assignment to interventions (e.g., participants, care providers, outcomes assessors), and how	9.20 ± 1.2	
11a1) Describe any changes to blinding, if applicable	8.68 ± 1.6	
11b) Describe any possible similarities between the interventions	7.79 ± 2.9	
Statistical methods		
12a) Describe the statistical methods used to compare groups for primary and secondary outcomes	10.00 ± 0.0	
12b) Describe the methods for additional analyses, such as subgroup analyses and adjusted analyses	9.85 ± 0.4	
12c) Describe the type of analysis used: intention-to-treat, complete-case, or per-protocol	9.45 ± 0.9	9.43 ± 1.1
12c1.1) If an intention-to-treat analysis was used, describe which imputation technique was applied to replace missing data	9.05 ± 2.1	9.43 ± 1.0
12c1.2) If per-protocol analysis was used, describe which protocol deviations occurred		9.00 ± 1.5
Results		
Participant flow (a diagram is strongly recommended)		
13a) For each group, describe the numbers of participants who were randomly assigned, received the intended treatment, and were analyzed for the primary outcome	9.80 ± 0.5	Omitted—identical to CONSORT.
13b) For each group, describe losses and exclusions after randomization, along with the reasons	9.45 ± 1.8	Omitted—identical to CONSORT.
13c) For each group, describe the percentage of compliance/adherence to the intervention	9.21 ± 1.9	8.80 ± 2.2
13c1.1) Describe whether adverse events, such as injuries occurred, including the number of events, dropouts, and reasons for dropouts	9.25 ± 2.0	8.97 ± 2.1
13c1.2) If technology was used, report adverse events, such as injuries, including the number of events and reasons for dropouts related to the technology		8.69 ± 2.2
Recruitment		
14a) Report the dates defining the recruitment and follow-up periods	9,20 ± 1,9	Omitted—identical to CONSORT.
14b) Report the reasons why the trial ended or was stopped	8,89 ± 1,9	
Baseline data		
15a) Provide a table showing baseline demographic and clinical characteristics for each group (cf. 6)	9.79 ± 0.4	9.7 ± 0.7
15a1) Report the mean or median age and age range for each group	9.21 ± 2.1	
15a2) Report the standard deviation of the mean age for each group.		9.2 ± 1.6
Numbers analyzed		
16) For each group, report the number of participants (denominator) included in each analysis and whether the analysis was conducted according to the originally assigned groups	9.50 ± 0.8	Omitted—identical to CONSORT.
Outcomes and estimation		
17a) For each primary and secondary outcome, report the results for each group, including the estimated effect size and its precision (such as 95% confidence interval)	9.45 ± 1.1	
17b) For binary outcomes, present both absolute and relative effect sizes (recommended)	9.53 ± 0.8	
Ancillary analyses		
18) Report the results of any other analyses performed, including subgroup analyses and adjusted analyses, distinguishing pre-specified from exploratory analyses	9.50 ± 0.8	
Harms		
19a) Describe all important harms or unintended effects in each group (for specific guidance, see CONSORT for harms)	9.60 ± 0.8	
19a1) Describe whether adverse events, such as injuries, occurred, including the number of dropouts and the reasons for dropouts	9.11 ± 2.1	9.17 ± 1.8
19a2) If technology was used, report adverse events, such as injuries, including the number of events and reasons for dropouts related to the technology	9.10 ± 2.1	8.93 ± 1.9
Discussion		
Limitations		
20a) Describe trial limitations, addressing sources of potential bias (e.g., heterogeneity of characteristics, differences in baseline characteristics, changes in blinding), imprecision, and, if relevant, multiplicity of analyses	9.20 ± 1.6	Omitted—identical to CONSORT.
20b) Describe unexpected changes to the overall study protocol	9.10 ± 2.0	
20c) Describe whether and how the results answer the RQ	8.70 ± 2.3	
20 d) Discuss imprecision and relevant differences in analyses or changes across groups	8.80 ± 2.2	
20e) Discuss whether surrogate markers adequately addressed the RQ	8.11 ± 2.4	
20f) Address all aspects of 5i)	8.25 ± 2.4	
Generalizability		
21) Report and discuss the generalizability (external validity, applicability) of the trial findings	9.28 ± 0.9	
21a) Report and discuss the effect sizes	8.40 ± 2.3	
Interpretation		
22) Provide an interpretation consistent with the results, balancing benefits and harms, and considering other relevant evidence	8.80 ± 1.9	
22a) Discuss all possible confounders that might affect the observed effect	8.20 ± 2.5	
Other information		
23) Registration: Include the registration number and the name of the trial registry	9.70 ± 0.7	
24) Protocol: If available, indicate where the full trial protocol can be accessed	9.15 ± 1.6	
25) Funding: Report sources of funding and other support (such as supply of drugs), the role of funders, and any support provided by training/software development companies	9.89 ± 0.5	

*“*Omitted—identical to CONSORT”. Note that some items were not sent to the editors because they remained unchanged from their CONSORT version. Mean ± SD

### Results of stage 5 – face-to-face expert meeting: discussion of guideline content

After reviewing the free-text responses, the group concluded that some aspects flagged as redundant should not be removed. Although certain items overlapped, it was agreed that they addressed distinct aspects of reporting and could offer valuable guidance for the future. Consequently, a guidance table was created to clarify the relevance of these items, providing justifications and examples for each. In the final stage (Stage 6), the table was integrated into the online questionnaire for the journal editors (see Supplemental Material 3).

### Results of stage 6 – online survey of editors (Consensus round 2)

After sending the final survey invitation to the identified editors (*N* = 311), 39 editors participated in the online survey (*n* = 31 valid responses). Overall, agreement ranged from 6.67 for item 3.2.05: Mean level and SD of education in each group, specify if years or degree (clarify but leave to author which to choose), to 9.77, for item 4.1.4: Duration of exercise (length of time spent on each exercise session in the intervention, in minutes), out of 10 points, underscoring the importance of standardized reporting.

The final checklist included in the guideline was rated by the editors, as shown in Table 2.

Significant differences between the ratings of the experts and editors were found for items 2b (F(1) = 5.416; *p* = 0.024; η² = 0.093), 5b (F(1) = 4.956; *p* = 0.031; η² = 0.094), 5e5 2b (F(1) = 4.149; *p* = 0.047; η² = 0.080), and 5e6 2b (F(1) = 7.150; *p* = 0.010; η² = 0.130), with higher ratings provided by the editors.

Moreover, the editors provided 50 comments (cf. Supplemental Material 3). Some of these comments, particularly those regarding abstract details related to word count and redundancies, overlapped with the comments from the PhysAgeNet Cost Action members (cf. Supplemental Material 2).

**Fig. 3:**
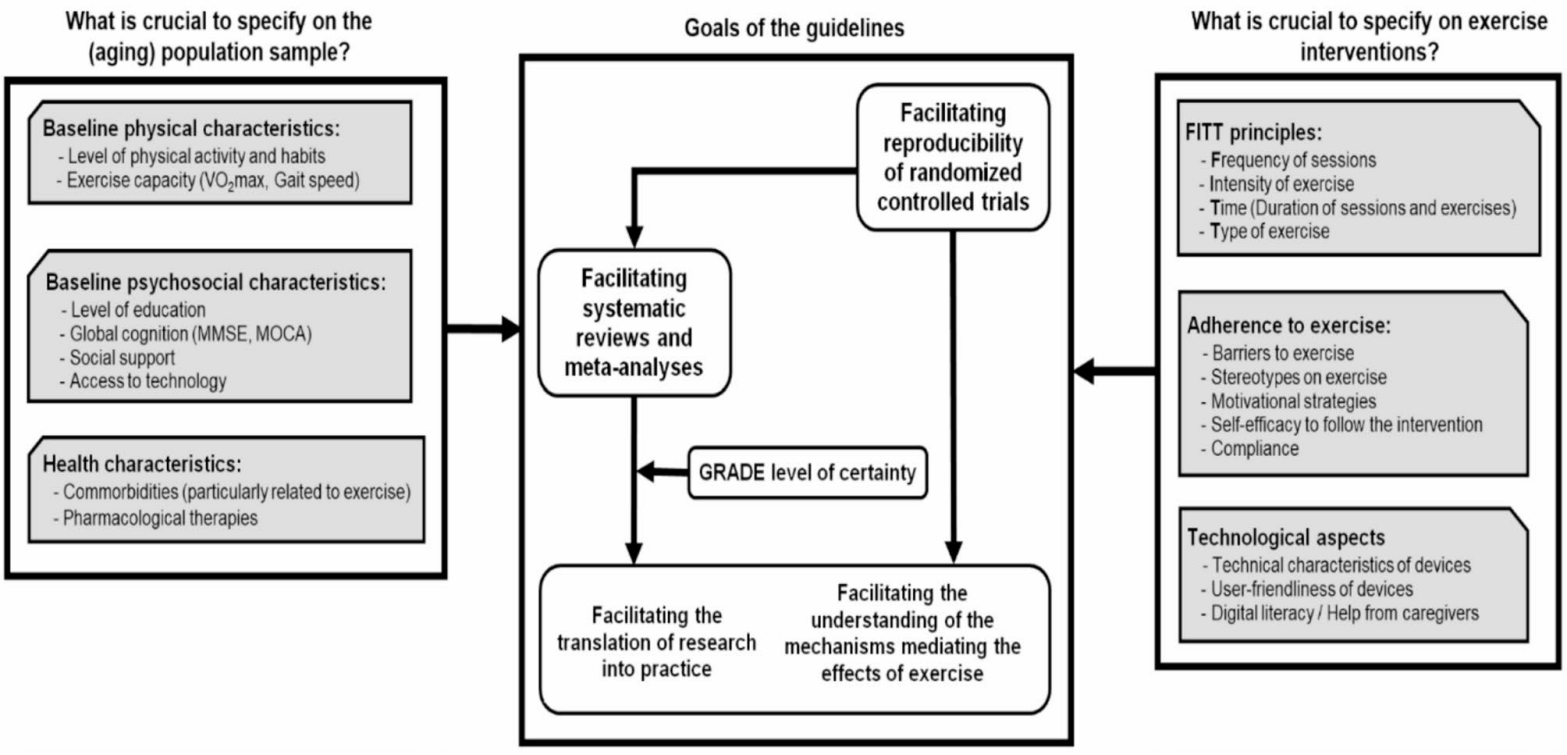
Final overview of the contextual factors of the PETIO guideline

The editors’ responses also addressed aspects of reporting participants’ characteristics and baseline physical fitness. Furthermore, they requested additional information on ethical approvals and graphical visualization. Altogether, the process led to the final guideline, which included all items and their explanations (cf. Supplemental Material 4). Fig. 3 provides an overview of the key considerations for reporting exercise intervention studies in older adults.

## Discussion

 Research has identified several barriers to implementing exercise interventions for older adults, including the lack of detailed protocols and inconsistent reporting of adherence and outcomes. These shortcomings hinder the reproducibility of interventions, reduce comparability across studies, and limit the applicability of research findings in real-world settings. 

Therefore, the aim of this guideline development was to enhance and standardize the reporting of exercise intervention studies for older adults. By ensuring that all relevant details are transparently and consistently documented, this guideline will facilitate the assessment of study quality, enable accurate replication, and strengthen the synthesis of evidence across studies. Ultimately, better reporting will advance the field of exercise science and contribute to improved health outcomes for older adults. To reduce persistent deficits in reporting despite the availability of existing checklists, authors are encouraged to systematically use the PETIO guideline as a complementary extension to CONSORT when designing and reporting exercise trials in older adults. Specifically, PETIO should be applied as a pre-submission checklist to ensure that critical intervention details, such as the rationale, dose (FITT principles), tailoring, adherence, and contextual factors, are explicitly described. Using the guideline during study design and manuscript preparation will help prevent omissions, improve transparency, and facilitate replication and meta-analysis.

Empirical evidence and the ongoing reproducibility crisis in science underscore the need for standardized, detailed, and transparent reporting of research methods and findings. Standardized reporting is particularly important for comparing the outcomes of different exercise interventions. Reproducibility is essential for validating their efficacy. Without structured reporting, critical details about the intervention - such as the type, intensity, duration, and frequency of exercise - may be omitted or reported inconsistently. This can lead to difficulties in replicating studies and verifying results [57].

### Reflection on the new guideline highlighting improvements or innovations

 Reporting guidelines in health research, such as CONSORT for randomized controlled trials, have improved transparency and consistency in scientific publications. However, in the context of exercise research, they are not without criticism. A common concern is that these guidelines are often too general and not tailored to the unique characteristics of exercise interventions, providing insufficiently detailed guidance for specific populations or study contexts [58]. For example, researchers working with older adults or individuals with multiple chronic conditions may find that certain aspects, such as physical or cognitive diversity, are not adequately addressed in existing guidelines. This means that although existing guidelines are comprehensive, their instructions are too general and lack the specificity required for particular populations (e.g., older adults) and specific types of intervention (e.g., exercise). These aspects have been addressed in the final version of the guideline provided here.

Additionally, some guidelines have not kept up with recent developments in health research. As fields such as digital health, remote interventions, and patient-centered approaches evolve, many existing guidelines lack clear recommendations for reporting these innovations. At this stage, the new guideline also addresses the current discussion on supporting technologies for exercise studies in older adults.

Furthermore, contextual factors, such as cultural differences, health system structures, or technological access, are often overlooked, even though they can significantly affect how interventions are delivered, implemented, and understood [59]. 

Since the completion of the PETIO guideline, updated versions of the SPIRIT and CONSORT checklists have been released [43, 45]. Notably, these updated guidelines introduced a new “Open Science” section that aligns with PETIO’s focus on transparent reporting. Most items in this section are already covered by the PETIO guideline, except for “data sharing” and “dissemination policy”, as these were previously included in the “Other information” section of earlier CONSORT guidelines. SPIRIT 2025 also introduced a “Patient and public involvement” section, which could be considered in future iterations of the PETIO guideline as exercise research increasingly moves toward participatory approaches [60]. PETIO covers aspects of physical exercise training in older adults that are not yet addressed in the SPIRIT and CONSORT 2025 updates. These include elements unique to exercise interventions, such as the FITT principle, the integration of technology, and age-related considerations.

### Integration of feedback into the development process

 The whole guideline development process was accompanied by several rounds of interdisciplinary discussion and feedback. The interdisciplinary panel demonstrated a generally high level of agreement across the evaluated items, with mean ratings ranging from 7.55 to 10.00. Regarding the new items added for exercise description, the lowest agreement was observed for item 5i2: “If applicable, report beliefs and stereotypes of participants concerning the effects of regular exercise on health”. However, the authors’ working group considered the item relevant and retained it in the PETIO guideline. Nonetheless, the result suggests that the item may pose challenges in terms of clarity, necessity, or applicability across various interdisciplinary research contexts. It may also reflect the complexity and variability of such psychosocial factors, which are often underreported or difficult for researchers to quantify in standardized formats. In contrast, item 12a: “Describe statistical methods used to compare groups for primary and secondary outcomes” received unanimous approval, reflecting both its fundamental importance and clear wording.

In addition to the quantitative ratings, panel members provided a total of 51 free-text statements, offering a range of qualitative insights. Several of these comments focused on abstract reporting, particularly regarding the clarity and completeness of information presented. A significant number of remarks addressed potential redundancies in the items, either with the existing CONSORT guidelines or among the newly proposed items, suggesting the need for more concise and non-overlapping content. Other feedback related to the level of detail and precision in the descriptions of the items in our proposal, with suggestions to improve specificity where necessary. Some panel members proposed the inclusion of new items not currently covered, while others commented on difficulties in understanding or interpreting certain items, highlighting opportunities to optimize language and phrasing. Additional observations covered broader or miscellaneous aspects that did not fit into the above categories, yet still contributed constructively to the development process. These qualitative inputs were taken into account and are presented in Supplemental Material 2.

Moreover, the editors involved in the process provided an additional 51 comments. Notably, some of their remarks overlapped with those made by the PhysAgeNet COST Action panel, particularly regarding the abstract, highlighting issues such as word count constraints and redundant phrasing. Beyond these shared concerns, the editors also emphasized the importance of reporting participants’ baseline characteristics, including their physical fitness levels at the start of the study. They highlighted the need for clearer documentation of ethical approvals and procedures and advocated for the inclusion of high-quality graphical visualizations to support the presentation of key findings. These comments, presented in Supplemental Material 3, were also taken into account in the development of the guideline.

Taken together, the quantitative ratings and qualitative feedback from both the interdisciplinary panel and the editors reflect strong overall endorsement of the proposed items, while also highlighting meaningful areas for refinement. The convergence of perspectives underscores the value of collaborative, cross-disciplinary input in ensuring clarity, relevance, and completeness in reporting standards.

- 5b) *Describe exercise physiology aspects (cardiovascular*,* metabolic*,* and muscular adaptations)*,* including a description of exercise progression (e.g.*,* increases in difficulty*,* duration*,* and frequency of the exercise program);*
- 5e5) *Whether the exercises are tailored or generic (one-size-fits-all); if tailored*,* describe how they were adapted to the individual;*
- 5e6) *Whether there are any simultaneous or consecutive exercise/intervention components (provide a brief*,* detailed description).*

This result suggests that editors may either possess greater experience in evaluating reporting quality or hold higher expectations regarding reporting standards within their journals. If the latter is true, it underscores the importance for authors to closely follow reporting guidelines when aiming to publish in high-quality journals.

### Suggestions for implementation and further development

The implementation of our newly developed reporting guideline for exercise interventions in older adults is expected to improve the clarity, completeness, and clinical applicability of study reporting in this growing research field. Piloting the guideline across multiple research groups would allow for an evaluation of its feasibility within existing research workflows and help identify its perceived value among end users. To assess user experience, a mixed-methods approach could be applied. Quantitative survey data might indicate whether most users find the guideline clear and useful, while qualitative interviews could provide deeper insights, highlighting the value of structured prompts for reporting adherence, adverse events, and participant feedback - elements that are often omitted in previous literature.

Initial feedback from the editors suggests that the guideline would enhance the structure of intervention descriptions, particularly in areas that are traditionally underreported, such as tailoring exercise to age-related limitations, implementing safety adaptations, and tracking participant adherence.

Training sessions and reference materials (e.g., templates, online presentations) might play a critical role in supporting guideline uptake. Therefore, as a next step, the additional supporting materials need to be discussed. Future directions might also include co-designing a digital implementation tool (e.g., an interactive checklist or form), expanding educational resources, and engaging a broader stakeholder group - including journal editors and older adult representatives - for further validation. Future work should also address the translation of the PETIO guideline into other languages, beginning with major European languages via the PhysAgeNet network.

PETIO is not designed to establish absolute thresholds of study quality. It was created as a reporting tool rather than a quality rating scale. Future work, based on empirical validation and potentially integrating GRADE or ROB2 frameworks, could help establish such thresholds. To enhance the scientific robustness of future versions of the guideline, the next iterations should also consider how reported interventions align with the strength and quality of the underlying evidence. In evidence-based medicine, and specifically for exercise interventions, the strength of evidence should be graded according to its quality. The GRADE approach provides a framework for evaluating evidence on clinical questions (e.g., how chronic exercise affects older adults’ health), taking into account factors such as study design, bias, inconsistency, imprecision, and effect size [61]. Evidence may be downgraded if these factors raise serious concerns, with risk of bias being particularly critical in exercise-related research.

Risk of bias in RCTs is often assessed using the ROB2 tool [62], which evaluates five domains: (1) bias arising from randomization; (2) deviations from intended interventions; (3) missing data; (4) outcome measurement; and (5) selective reporting. Domain 2 often indicates a high risk of bias in behavioral studies (e.g., exercise, cognitive behavioural therapy), particularly when blinding of participants and intervention providers is not feasible. In exercise trials, for instance, participants and intervention providers typically know the group assignments, therewith increasing the likelihood of bias. This is especially problematic if participants modify their behavior outside the assigned protocol due to dissatisfaction with their group allocation. Bias from such deviations can be minimized by: (1) tracking physical activity outside the structured intervention; (2) measuring participant preference prior to randomization; and (3) evaluating participant satisfaction after the intervention. These measures can help control contamination effects between groups. Domain 4 bias arises when outcomes are self-reported (e.g., mood, quality of life), particularly if participants are aware of their group assignment. To reduce this risk, external assessors, blinded to group assignment, can observe or interview people close to the participant. The development and use of improved assessment tools for the quality of reporting may further support unbiased outcome measurement in behavioral trials.

PETIO should be considered a complementary extension to CONSORT. When journals require the use of CONSORT as reporting guidelines, authors should complete the CONSORT checklist in full and are encouraged to integrate the PETIO items not covered by CONSORT, such as those addressing exercise and age-related aspects. This approach ensures compliance with journal policies and enhances transparency in exercise trials involving older adults.

## Limitations

Several limitations should be acknowledged in the development process and scope of the current guideline. First, although the items were developed through expert consensus and informed by established frameworks such as CONSORT, the guideline has not yet undergone systematic pilot testing. This represents a limitation in terms of practical applicability and usability in real-world research settings. However, it is important to note that the present work primarily adds physical exercise and age-specific aspects to the CONSORT framework, rather than proposing a complete overhaul. Future pilot studies will be essential to refine the guideline based on empirical feedback.

Secondly, the current guideline is primarily oriented toward quantitative research methods and does not comprehensively address qualitative methodologies, such as ethnographic approaches or studies focused on individuals’ lived experiences and perceptions. This focus may limit its applicability to research designs that explore more nuanced or subjective aspects of health and aging.

Lastly, the guideline has been developed with a broad scope in mind and does not address the specific reporting needs of studies targeting particular pathologies in older adults. Future adaptations or extensions may therefore be necessary to tailor the recommendations to specific clinical populations or research contexts.

## Conclusion

Adopting the PETIO guideline for physical exercise intervention studies in older adults is expected to enhance the transparency, quality, and completeness of research reporting in this field. The high level of agreement among journal editors in the final round confirms the relevance of this guideline for future research publications in the target journals. The guideline can also support researchers in designing and reporting interventions, help reviewers and editors assess the quality of manuscripts submitted for evaluation, and assist policymakers in implementing effective interventions at larger scales. Like other guidelines, the current version will evolve over time as new evidence, innovative supporting technology, or usability research emerges.

## Supplementary Information

Supplementary Material 1

Supplementary Material 2

Supplementary Material 3

Supplementary Material 4

## Data Availability

At this stage the data will be available at the corresponding author until the PhyAgeNet will decide how to make any data available.

## Footnotes

1 In describing the aging population, the WHO refers to people aged 60 years and older (WHO, 2024).
