# Supplementary material for: Reporting randomised trials of physical exercise or training interventions in older adults: the PETIO guideline
Source: Eur Rev Aging Phys Act. 2025 Dec 2;22:24. doi: 10.1186/s11556-025-00390-x (PMC12670822; doi:10.1186/s11556-025-00390-x)
Supplement: Supplementary file 1 — Supplementary Material 1 [file 11556_2025_390_MOESM1_ESM.docx]

**CONSORT reporting guidelines extension –**

**Editors catalog of questions for**

By CA 20104 - Network on evidence-based physical activity in old age (PhysAgeNet)

**Question 1:**

Participating experts rated the following aspects for improving abstract reporting for exercise and PA studies with older adults as important or very important. Do you agree? (Rate from 1 (totally disagree) to 10 (totally agree).

1. **Additional information within the abstract next to CONSORT guidelines**

- Include a structured summary of trial design, methods, results, and conclusions (for specific guidance see CONSORT for abstract)
- Describe the population (healthy, diseased, etc.)
- If the title cannot integrate the PICO scheme (Population-Intervention- Comparison-Outcome) according to a word limit, report all PICO criteria in the abstract
- Report age (mean > xx years; age range)
- Report number of males/females
- Report information about the FITT principles in exercise studies

**Open Question 1a:**

Where did you disagree and why?

**Open Question 1b:**

Do you want to give any additional recommendation?

**Question 2:**

Participating experts rated the following aspects for improving the reporting within the introduction for exercise and PA studies with older as important or very important. Do you agree? (Rate from 1 (totally disagree) to 10 (totally agree).

1. **Additional information within the introduction/ theoretical background next to CONSORT guidelines**

- Describe potential theoretical models or mechanisms (including summary of previous studies) explaining why the proposed intervention might work and how they are related to the outcome of interest (e.g. cardiovascular adaptations or increase of muscle mass, etc.)
- Include specific objectives or hypotheses
- Formulate the research question and hypothesis according to the outcomes of interest
- Justify if the outcomes match the research questions

**Open Question 2a:**

Where did you disagree and why?

**Open Question 2b:**

Do you want to give any additional recommendation?

**Question 3:**

Participating experts rated the following aspects for improving the reporting within the methods for exercise and PA studies with older as important or very important. Do you agree? (Rate from 1 (totally disagree) to 10 (totally agree). Part I

1. **Additional information within the methods next to CONSORT guidelines**

- Describe trial design (such as parallel, cluster, factorial) including allocation ratio
- Describe changes to methods after trial commencement (such as eligibility criteria), with reasons

Regarding participants description (for all intervention studies regarding older adults)

- Report eligibility criteria for participants, including:
  - Mean age or median of sample age depending on the distribution of your sample, and age range
  - Inclusion/exclusion criteria
- Provide a table comparing the main characteristics of the different groups of participants, including:
  - PA level at baseline” and end of intervention; change to “PA
  - level/functional capacity at baseline”
  - percentage of women in each group
  - Mean level of education (and SD) of the participants in each group, specify if years or degree; clarify but leave to author which to choose
  - Health status (objective and/or subjective health?)
  - If the study design includes technology support or usage, include:
  - Experience with technology
  - Participants' access to the technology
  - Digital literacy
  - Aspects of tailoring the technology
- Describe settings and locations where the data was collected

**Open Question 3a:**

Where did you disagree and why?

**Open Question 3b:**

Do you want to give any additional recommendation?

**Question 4**

Participating experts rated the following aspects for improving the reporting within the methods for exercise and PA studies with older as important or very important. Do you agree? (Rate from 1 (totally disagree) to 10 (totally agree). Part II

Regarding the description of the intervention (for all intervention studies regarding older adults)

- Describe the interventions for each group with sufficient details. If multimodal, provide details for every modality to allow replication, including how and when they were actually administered., including a description of:
  - Exercise type (single mode or multimodal)
  - Exercise frequency (number of sessions per week)
  - Duration of exercise (length of time spent on each exercise session in the intervention (in minutes))
  - Exercise intensity (level of difficulty or effort exerted during the exercise in the intervention and methods used to assess and monitor it, e.g., integrate the percentage of HR max/HR reserve/VO2max/VO2peak; RPE/fatigue before and after intervention/report also the scale (i.e. 6-20, 1-10))
- Describe exercise physiology aspects (cardiovascular, metabolic, and muscular adaptations), including a description of:
  - Exercise progression (increase in the difficulty, duration, and frequency of the exercise program)
  - Intensity changes in different exercise types
  - Resting times (duration of rest or recovery between sets or exercises during the intervention)
  - METs for exercise program/its components: This refers to the estimated energy expenditure during physical activity intervention
  - If technology was used, describe if and how exposure is controlled in the technology
- Describe whether there is any non-exercise component
- Describe exercise settings, including a description of:
  - Whether exercise is performed individually or in a group
  - Whether exercise is supervised or unsupervised and how exercise was monitored
  - Any home program component
  - Whether the exercises are tailored or generic (one-size fits all): If tailored, describe how it was tailored to the individual
  - Whether there are any simultaneous, consecutive exercise/ intervention components (add a short detailed description)
  - Type of exercise equipment
- If technology is used, describe how
  - exercise was monitored (e.g.,telemonitoring)
  - The design, specific functions (including a list of examples with description), software, interface, adaptation methods, algorithms
  - progression is programmed
  - How the outcome is calculated if available
  - Specific match between technology of intervention and outcome

**Open Question 4a:**

Where did you disagree and why?

**Open Question 4b:**

Do you want to give any additional recommendation?

**Question 5:**

Participating experts rated the following aspects for improving the reporting within the methods for exercise and PA studies with older as important or very important. Do you agree? (Rate from 1 (totally disagree) to 10 (totally agree). Part III

- Describe type of control group in detail (e.g., was the control group active or passive? If active, what type of activity did they perform?)
  - Report whether the control group was blinded

Describe if and how exposure is controlled for the control group in the technology if technology was used

- Describe motivational control aspects
  - Describe how compliance/adherence to exercise is
  - measured/assessed
  - Describe of motivational strategies and behavioral change techniques if used
  - If applicable, how do participants get compensated for study participation
- Describe the extent to which intervention was not delivered as planned if applicable

**Open Question 5a:**

Where did you disagree and why?

**Open Question 5b:**

Do you want to give any additional recommendation?

**Question 6:**

Participating experts rated the following aspects for improving the reporting within the methods for exercise and PA studies with older as important or very important. Do you agree? (Rate from 1 (totally disagree) to 10 (totally agree). Part IV

- Control for confounding factors
- If applicable, report beliefs and stereotypes of participants concerning the effects of regular exercise on health
- Physical activity (PA) practiced by the participants beside the intervention. The method to measure PA level should be mentioned (e.g., actimeter, questionnaire)
- If applicable, report participants’ preference of the different groups for a specific intervention (particularly when several interventions are implemented)
- Describe type of analysis used: intention-to-treat, complete-case, perprotocol
  - Describe if the intention-to-treat analysis was used, which imputation technique was carried out to replace missing data,
  - if per-protocol analysis, what protocol was not followed
- Describe for each group, percentage of compliance/adherence to the intervention
  - Describe if adverse events like injuries occur, number, dropouts, and

reasons for dropouts, this also has to be reported if technology was used

**Open Question 6a:**

Where did you disagree and why?

**Open Question 6b:**

Do you want to give any additional recommendation?

**Question 7:**

Participating experts rated the following aspects for improving the reporting within the results for exercise and PA studies with older as important or very important. Do you agree? (Rate from 1 (totally disagree) to 10 (totally agree). Part III

1. **Additional information within the results next to CONSORT guidelines**

- Report mean age or median and age range per group
- Describe if adverse events like injuries occurred, number of dropouts, and reasons for dropouts
- If technology was used, report on adverse events like injuries, number and reasons for dropouts according to the technology

**Open Question 7a:**

Where did you disagree and why?

**Open Question 7b:**

Do you want to give any additional recommendation?
