# Supplementary material for: Reporting randomised trials of physical exercise or training interventions in older adults: the PETIO guideline
Source: Eur Rev Aging Phys Act. 2025 Dec 2;22:24. doi: 10.1186/s11556-025-00390-x (PMC12670822; doi:10.1186/s11556-025-00390-x)
Supplement: Supplementary file 2 — Supplementary Material 2 [file 11556_2025_390_MOESM2_ESM.docx]

**Supplemental Material 2: Qualitative Comments of the Questionnaire: COST Members**

| **A** | **Abstract:** The following items are suggested to extend the CONSORT guidelines for reporting randomized controlled trials (RCTs) on exercise interventions in older adults. Please rate on the Likert scale from 1 (not agree) to 10 (fully agree) whether the item should be included in the description tool. | *1* | On some continents (for example Africa) duration of life is lower if compare with European countries.  So older adults are not from 60 years old, but from 50-55. How can this issue be incorporated in the abstract? |
| --- | --- | --- | --- |
|  |  | *1* | Information about the FITT principles is too nuanced/detailed to expect that it can be included in the abstract. Authors would need to write it in such a brief way that it would not really serve a purpose. |
|  |  | *1c* | Report age (mean >60 years; age range) - this should be the exact age of the participants rather than the inclusion criteria. I wouldn't define the constructs of 'older age' here, as that should be within the methodology or perhaps even introduction instead. |
|  |  | *1d, 1e* | I think these points are very important. However, I am concerned that providing detailed information will probably make the abstract too long (as required by most journals). |
| **B** | **Background and Objectives:** Please rate on the Likert scale from 1 (not agree) to 10 (fully agree) if, from your point of expertise, the single items should be included in the description tool for reporting interventional studies in older adults to extend the CONSORT guidelines. | *2a1* | *Too subjective - a reviewer might disagree with the mechanisms or theory described and whether or not they are well justified. Therefore, it should not be used as a checklist item. I think this is also why such detail is not given in the main CONSORT checklist.* |
|  |  | *2b1, 2b2* | Away from the phrasing typically used for a reporting checklist and are rather giving guidance on how to report, rather than what to report. Again, similar to the point above, these are also very subjective and are therefore not very appropriate for a standardized reporting checklist. |
|  |  | *2b2* | If the outcomes (or indicators of outcomes) are related to theoretical model (point 2a1), and they should be (!), point 2b2 is rather unnecessary. It should somehow be included/covered in point 2a1.  Justify if the outcomes match the research questions = It is not clear to me what is meant with this. |
| **C** | **Methods:** Please rate on the Likert scale from 1 (not agree) to 10 (fully agree) if, from your point of expertise, the single items should be included in the description tool for reporting interventional studies in older adults to extend the CONSORT guidelines.  **4 – Participants** | *General* | Possibly, risk of intervention should be evaluated for each participant. |
|  |  | *4a1* | Important, but would typically be reported in the results, not the methods, in a RCT. |
|  |  |  | You want average value in an abstract so is it OK to give possibility to report mean OR median age? Even if the distribution is not normal it could be provided mean AND median. |
|  |  | *4a2* | Redundant - "eligibility criteria" in 4a includes inclusion and exclusion criteria (see original CONSORT statement guidance). |
|  |  | *4b all items* | In my opinion, for a general reporting guideline, many of these items are far too specific and will not be applicable in many cases. For example, not all exercise studies will have PA levels as an outcome since it may be irrelevant to the goals of that specific study (not all exercise trials are trying to influence PA levels - some are looking at balance, some are looking at muscle strength, etc.). Of course, information on PA levels is nice to have in all cases, but it is subjective whether it is a critical outcome in all trials with older adults. Again here, it seems like you want to give guidance on optimal trial design, when a reporting checklist should focus not on mandating outcomes but on clear reporting of the outcomes chosen by the authors. I would advise mentioning all of these aspects in the guidance paper so that authors know what to report in the participant information section when applicable without actually including many potentially non applicable items on the checklist. This is also how the original CONSORT statement and checklist is arranged - the checklist does not mandate outcomes. |
|  |  | *4b3* | Since we make the effort to recommend refined reporting guidelines, we may as well try to "enforce" standardized reporting in such an important outcome as education. I would vote to propose years of education as absolute preferable reported metric tool for reporting interventional studies in older adults to extend the CONSORT guidelines. |
|  |  | *4b6* | Access to tech will be connected to the experience with tech. |
|  |  | *4b7* | Nice but what is the concept of quantification of this variable? |
|  |  | *4b8* | It seems more suitable to methods description.  Potential, additional confounding factors:   - initial level of physical fitness (especially if the outcomes are connected to physical fitness or improvement of functionality), - the season of the year during which intervention is provided (it might modify current PA level as well as adherence to the intervention). |
|  |  |  |  |
|  | **5 – Interventions** | *5* | The structure needs improving here since the items are not logically organized (i.e., items on technology are scattered throughout). |
|  |  | *5a4* | Good to include but the text is too detailed. Remove everything from "e.g.," onwards. |
|  |  | *5b* | Not necessary to understand the subpoints - I suggest removing. |
|  |  | *5b2* | Redundant when including 5b1. |
|  |  | *5b4* | Too specific and not applicable to all types of interventions - remove.  Seems to be covered by 5a4. |
|  |  | *5c* | Too vague. |
|  |  | *5e4* | Redundant since 4c already asks for this. |
|  |  | *5e3* | Redundant since 5e2 already asks how exercise was monitored. |
|  |  | *5e6* | Probably redundant since previous points should already result in this being reported. |
|  |  | *5e8-11* | Not clearly described but perhaps in too much detail. Consider combining into one point that asks authors to report these multiple aspects of the technology used, when applicable. |
|  |  | *5f* | Seems incomplete - there are many other ways to categorize control groups other than just active or passive. Placebo, nocebo, matched on various aspects or not, etc. I think this can be briefer in the checklist with more detail in the associated guidance text. In case of tech usage in PA passive control group could not be enough. There would be no possibility to answer the question if the potential effect in dependent variable is connected to tech or to PA (two independent variables) - in other words, control condition is cut-off. In the best case it should be 4 groups model (active, passive, active with tech, active without tech). On the other hand, if the control group is active it seems to be very important to report if the level of total volume of PA (FITT) is similar to experimental group. |
|  |  |  | Mostly control group in PA intervention cannot be blinded (in consent form participants receive information about intervention). Maybe it is possible for waiting list or active control group. |
|  |  | *5f1* | Less detailed compared to the original CONSORT checklist item on blinding. It should also be reported if the intervention group was blinded, if the people delivering the intervention were blinded to the group assignment of the participants, as well as the people performing the assessments and analyses. |
|  |  | *5f2* | Redundant (same as 5b5 - though the phrasing is better in 5f2). |
|  |  | *5g* | Too vague. |
|  |  | *5g2* | Too specific for a single checklist item. |
|  |  | *5g3* | It might be important. However, there is the basic question if they should be! |
|  |  | *5h* | Poor grammar makes it difficult to understand exactly what should be reported. |
|  |  | *5i* | These are very detailed and specific and will not be applicable to all studies and therefore should not be reporting checklist items. Consider only mentioning them as recommendations in the text. |
|  |  | *5i2* | it's important. However, participants should be rather encouraged not to engage in other activities (especially those which might influence the effect of intervention) - it might be very difficult to control this confounder. |
|  | **11 – Masking** | *11* | Makes the blinding item in the intervention section redundant. |
|  | **12 – Statistical methods** | *12c* | Phrasing is quite unclear. |
|  | **13 – 19 – Participant flow (a diagram is strongly recommended)** | *13c1* | Redundant - this is covered by the harms section of CONSORT. |
|  |  | *15* | Baseline data makes a lot of the "Participant Information" items on the previous page redundant. This is also why I think the additions in the participant information are confusing - they both mandate outcomes and are in addition to section 15 which already asks for relevant demographic and clinical characteristics. All we really need is in the text guidance to emphasize which characteristics are important to consider in exercise trials with older adults, rather than adding a huge number of potentially redundant or not applicable items to the reporting checklist. |
|  |  | *19a1, 19a2* | Redundant - the CONSORT harms part covers those. |
|  |  | *Recruitment* | These items are important. However, I'm not sure if they belong to the result section. |
| **D** | **Limitations:** Please rate on the Likert scale from 1 (not agree) to 10 (fully agree) if, from your point of expertise, the single items should be included in the description tool for reporting interventional studies in older adults to extend the CONSORT guidelines. | *20b* | Redundant - the original CONSORT items already ask for changes in methods and outcomes. |
|  |  | *20c,d,e,f* | These items are all subjective and not factual, so they should not be included on a standardized reporting checklist. Consider discussing these aspects in the guidance text instead. |
|  |  | *20f* | As mentioned at section 5, these items are too specific for a reporting checklist. Place this information in the guidelines text as advice, not as a standardized checklist item. |
|  |  | *21a* | Specific guidance without necessary context. Whether and in what way effect sizes should be discussed is greatly dependent on the nature of the statistical approach which again is not something that should be mandated by a reporting checklist. The results section already requires reporting of effect sizes - whether or not they are discussed in the discussion is scientifically irrelevant since the interpretations of the authors may greatly differ from the readers. |
|  |  | *22a* | Redundant - this is covered by the CONSORT text for 22. |
