# Supplementary material for: Reporting randomised trials of physical exercise or training interventions in older adults: the PETIO guideline
Source: Eur Rev Aging Phys Act. 2025 Dec 2;22:24. doi: 10.1186/s11556-025-00390-x (PMC12670822; doi:10.1186/s11556-025-00390-x)
Supplement: Supplementary file 3 — Supplementary Material 3 [file 11556_2025_390_MOESM3_ESM.docx]

**Supplemental Material 3: Qualitative Comments and Questions: Editors**

| **A** | **Additional information within the abstract next to CONSORT guidelines** | *General comments* | These are all of importance but a number of journals have a word limit for abstracts. All of this information is required but may not necessarily have to be in the abstract.  I am not sure that the full PICO needs to be included in the abstract, as it may make abstracts way too long.  The FITT information this can be elaborated upon elsewhere. Other information (PICO) is more important.  These elements should all be in the abstract. It is the only component of a manuscript that many people will read.  Any safety issues of adverse events should be in the abstract  While it is important for such detail to be in the article, it does not necessarily need to be in the abstract. |
| --- | --- | --- | --- |
|  |  | *1.5* | Sex can be in the body of the paper, just the total n in the abstract |
|  |  | *1.6* | This is too much information for most journal abstract word limits and is better off in the manuscript. The abstract could say it was aligned with ...... |
| **B** | **Additional information within the introduction/ theoretical background next to CONSORT guidelines** | *General comments* | The outcomes matching research questions should be detailed in relation to the methodological description.  The last point of matching outcomes and questions can be in the materials and methods section. |
|  |  | *2.3* | I think this sub-point largely duplicates the previous one. |
|  |  | *2.4* | I am not sure that the introduction is the right place for these guidelines. |
| **C** | **Additional information within the methods next to CONSORT guidelines** | *General comments* | Be aware of ageism. Tailoring or digital literacy should be reported similarly in trials in younger and older adults. Not doing so would be based on the idea that older persons cannot cope with technology.  The population should be described but a table with details about the characteristics can also be part of the results.  I agree with technology but it is only relevant if technology was used in the intervention delivery process.  I don’t think access and experience with technology will be significant for all studies.  Agree for all as important to understand the utilization and context of the technology.  In older persons, baseline functional assessment (physical performance, frailty, dependence for ADLs), cognitive status and nutritional status are needed to understand results. Otherwise, there might be discrimination in access or selection bias, by non-disclosure of exclusions (typical, for instance, in those with cognitive decline).  The % of women is necessary only if both sexes are included in the study. |
|  |  | *3.2.1* | This should be in the results, not the methods. |
|  |  | *Section b* | This should all be in the results. |
| **D** | **The description of the intervention** | *General comments* | Using METS is based on the need of cardiovascular exercise. This does not seem to make sense in other types of exercise (i.e. balance training). Moreover, measuring METS in older persons is inaccurate or needs equipment.  The one related to METS as this is less secure and applicable across all groups - fidelity is important such as was the required programmed intensity actually reached. In other words, was data collected around the intensity to ensure the participant hit the required threshold for the intensity of the exercise [however it was measured].  I don’t disagree; however, many studies aren’t able to include VO2 max due to resource limitation so that may not be appropriate for resource limited settings.  The inclusion of METS is not always accurate. The description as described in other questions is better.  This is a lot of information and in many cases will need to be in an appendix or supplemental file. It doesn't matter where it is as long as it is available and transparent.  When technology is used, for editor it is important that name, type of product and producer are named. Afterwards there is no extra need to explain how it works, unless validation of technology is the main aim of article.  All program variables need to be discussed including cadence or velocity, exercises and exercise order, repetitions, loading, etc.  Technology seems to be used as a broad term here (and other questions). I am unsure what is meant specifically by technology. |
| **E** | **Details of the control group** | *General comments* | I would be cautious with compensation for participants.  In a recent meta-analysis, we found that descriptions of motivational strategies and behavioral change techniques are naïf and not based on strong psychological grounds. I understand that the psychological theoretical framework of such strategies (psychology has advanced greatly in recent years) needs also to be mentioned.  Fidelity should also be reported.  Consider clear statement of funding especially if from a business or product manufacturer. |
|  |  | *5.1.2* | Is not usually possible for many trials, which is fine as long as the assessor is blinded. |
| **F** | **Control for confounding factors** | *General comments* | Beliefs and stereotypes are cumbersome to measure and understand.  Nutrition is basic for exercise and is a stronger confounder than PA or preferences (or beliefs). This is specially so in older patients. Nutritional status and use of nutritional supplements is a confounder that should always be considered and reported, in my view. |
|  |  | *6.1* | Depends on the trial design - RCTs control for a lot of this. |
| **G** | **Additional information within the results next to CONSORT guidelines** | *General comments* | Consider reporting median and IQR, when appropriate (for example age).  The adverse events must become compulsory to report as it will help generate other statistics. Any other information to help economists calculate the cost-benefit of exercise interventions could be crucial and should be considered i.e., what is the estimated cost of the intervention.  Why do you mention injuries and not falls as adverse outcome? In older adults one would expect falls to be always reported as potential AE. |
|  |  | *7.2* | Better to report interquartile range for the mean age per group. |
| **H** | **Regarding your journal's policies and publishing practice: Which additional recommendation would you like to give us to further improve our guidelines?** | *Editor 1* | Transparency and reproducibility; Require the publication of raw data, code, and analysis plans in publicly accessible repositories.  Encourage pre-registered studies and the publication of study protocols to enhance credibility.  Reporting on AI and digital tools; Introduce a section on the use of AI and machine learning in data analysis or patient recruitment.  Require transparent disclosure of AI-assisted writing tools used in manuscript preparation. |
|  |  | *Editor 2* | The ethical approval should be part of the guidelines as well as reporting the trial registration and possible publication of the study protocol. These are forgotten too often in manuscripts I see on my editor table. |
|  |  | *Editor 3* | Our guidelines are incredibly comprehensive and if fully followed would lead to the ideal paper. However, research is not always straightforward and, in some cases, not all these criteria will be met. Key for me is that the guidelines are presented as good practice but a recognition that not every criterion will always be met. |
|  |  | *Editor 4* | I think these recommendations are great, and should always be reported. Pragmatically, I also know that it is difficult to monitor all of these parameters, so I would not automatically rate a study as low quality if some of these issues were not recorded and reported. |
|  |  | *Editor 5* | Anything on best practice for statistical presentation and any visual presentation of the data would be helpful. |
|  |  | *Editor 6* | Much of what you are after is already in place in various guidelines. I see you didn't mention the TIDieR guidelines in your intro, but had the CERT. |
|  |  | *Editor 7* | This guideline is very much for quantitative research methods. We do not focus on quantitative research methods, but ethnographic data, people lived experience and/or their perceptions |
|  |  | *Editor 8* | Recommendations should be as brief as possible. There are too many in this survey. |
|  |  | *Editor 9* | I think reporting all details of methods is important for reproducibility. |
|  |  | *Editor 10* | To provide a clear exercise background of the participants. |
