# Supplementary material for: Reporting randomised trials of physical exercise or training interventions in older adults: the PETIO guideline
Source: Eur Rev Aging Phys Act. 2025 Dec 2;22:24. doi: 10.1186/s11556-025-00390-x (PMC12670822; doi:10.1186/s11556-025-00390-x)
Supplement: Supplementary file 4 — Supplementary Material 4 [file 11556_2025_390_MOESM4_ESM.docx]

**Reporting randomised trials of Physical Exercise or Training Interventions in Older Adults: the PETIO guideline description tool**

|  | **Section and criteria** | **Reason for relevance** | **Example on how to report in your manuscript** |
| --- | --- | --- | --- |
| **1** | **Title and abstract** | | |
| 1a | Identification as a randomized trial in the title; if possible, follow the PICO scheme | - Relevant for searches in databases | **Example a:** An RCT on 12 weeks of cognitive, motor or combined cognitive-motor exercise to improve dual task walking in older adults: The role of baseline cognitive and motor fitness  **Example b:** Specify the target group: if the study focuses on “older adults diagnosed with mild cognitive impairment (MCI), use a title such as “Effects of resistance training on memory in older adults diagnosed with mild cognitive impairment: A randomized controlled trial.” |
| 1b | Structured summary of trial design, methods, results, and conclusions | - Relevant for searches in databases - Provides an overview of the main aspects of the article | **Trial design:** Parallel-group randomized controlled double-blind trial  **Methods. Participants:** Healthy older adults; data collected at the University of Chemnitz and the University of Münster  **Interventions:** Cognitive training (cogT), motor training (motT), cognitive-motor dual-task training (DTT)  **Objective:** This RCT examined whether baseline cognitive fitness (cf) and motor fitness (mf) moderates training effects of these interventions on cognitive-motor dual-task performance in older adults.  **Outcome:** Cognitive motor performance assessed at pre- and post-test using a Serial Threes task (S3), a Stroop task (STR), and a walking task. Outcomes were assessed under single-task (ST) and dual-task conditions (DT).  **Randomization:** Randomization was conducted at a 1:1:1 ratio using a computer-generated random allocation schedule, **Blinding:** parallel-group randomized controlled double-blind trial  **Results:** Number randomized: 128 participants were randomized in three groups (cogT, motT and DTT). Number analyzed: Participants (N = 97, aged 65–75) completed 12-week interventions in cognitive, motor, or cognitive-motor dual-task training. Outcome: In summary, linear mixed model results indicated that for both S3 as STR, cogT and DTT led to greater increases in cognitive performance than in motor performance across both ST and DT conditions, while the motT showed greater increases in motor performance than in cognitive performance. The results also showed that mf and the interaction between cf and mf did not moderate pre-post changes in cognitive or motor performance. However, cf did play a significant moderating role for the S3. When comparing the groups, in particular, cogT and motT showed opposing effects (t = 5.35). For individuals with higher cf, motor performance increased more in the cogT than in the motT. However, their cognitive performance increased more in the cogT than in the motT.Harms  **Conclusion, Trial registration:** This trial was retrospectively registered at…., **Funding:** The study was funded by the XX (grant XX) |
| *1b1* | Describe the population (e.g., community dwelling/healthy, diseased etc.) In the case of diseased population, mention the disease. | - To be consistent with other reporting guidelines it should be referred to the CONSORT guidelines | **Example a:** This example abstract title was: “An RCT on 12 weeks of cognitive, motor or combined cognitive-motor exercise to improve dual task walking in older adults: The role of baseline cognitive and motor fitness”. The abstract mention “healthy older adults”  **Example b:** If the title is “Strength Training in Older Adults: A Randomized Trial,” the abstract should explicitly mention: “Population: Community-dwelling older adults aged 65+; Intervention: 8-week progressive resistance training program; Comparison: Routine daily activities; Outcomes: Changes in lower-body strength and balance |
| *1b2* | All PICO criteria must be reported in the abstract if not specified in the title. | - Ensures clarity and transparency of key details (PICO) even if the title cannot fully capture them due to length restrictions |  |
| 1c | Reporting of the age (mean >60 years; age range) | - Ensures that study follows WHO definition of older age - Ensures the reporting of age range if relevant for interpreting the results | Participants aged 65–75 years (mean age = 69.44, SD = 3.81) |
| 1d | Reporting the number or percentage of males/females in the total sample. | - Important for the generalization and interpretation of results (i.e. according to distribution) | **Example a**: Of the 128 participants, 68 were females and 60 were males.  **Example b**: Of the 150 participants, 90 (60%) were female, and 60 (40%) were male. |
| 1e | Reporting of frequency, intensity, time and type of the exercises according to the F.I.T.T. principles. | - Important for assessing training quality and interpreting dose-response relationships. - Ensures consistency, transparency, and completeness of reporting. | **Example a:** Participants completed 12-week interventions consisting of two 60-minute sessions per week in one of three conditions: cognitive training (cogT), motor training (motT), or cognitive-motor dual-task training (DTT), all conducted at intensities below 60% of maximum heart rate (HRmax).  **Example b:** Participants performed resistance exercise using dumbbells, three times per week, using 60–70% of their one-repetition maximum, for 30-minute sessions during 3 months. |

| **2** | **Introduction** | | | | | | | |
| --- | --- | --- | --- | --- | --- | --- | --- | --- |
| 2a | Scientific background and explanation of rationale | | | | | | | |
| *2a1* | Description of the potential theoretical models or mechanisms (including summary of previous studies) | | - Provides a review of the literature including potential theoretical models or mechanisms explaining how the intervention may influence the outcome (cardiovascular adaptations, increase muscle mass...) - Contributes to the state of the art on dose-response relationships - Clarifies the conditions/ training   regimes/characteristics under which the intervention contributes to beneficial adaptations or positive results for older adults. | | | Evidence from studies that used moderate intensity aerobic exercise training suggests enhanced cardiorespiratory fitness (provide a reference; author, year). Specifically, aerobic exercise using a bicycle, 3–4 times per week for 6 months, at an intensity of 70–80% of VO₂max and with each session lasting 30 minutes, can increase cardiorespiratory fitness through physiological adaptations in the cardiovascular, respiratory, and musculoskeletal systems. In particular, studies have shown that training with these characteristics can improve stroke volume and myocardial efficiency, induce beneficial vascular adaptations such as reduced arterial stiffness and improved endothelial function, enhance blood flow, increase mitochondrial density, improve autonomic nervous system activity, among other effects (provide a reference; author, year). | | |
| *2a2* | Summary of previous studies (referring to the FITT principles) | |  |  |  |  |  |  |
| 2b | Specific objectives or hypotheses | | | | | | | |
| *2b1* | Formulate the research question and hypothesis according to the outcomes of interest and justify if the outcomes match the research question. | | - Important to justify the methods and statistical analysis | | | **Example a**: Specifically, we investigated whether baseline cognitive and motor fitness modulated the benefits of cognitive, motor and cognitive-motor dual-task training in improving cognitive-motor dual-tasking. More specifically, we hypothesized that cognitive-motor dual-task performance in persons with low baseline cognitive fitness benefited more from cognitive training than from motor or cognitive-motor dual-task training (H1). We further posited that cognitive-motor dual-task performance in persons with low baseline motor fitness benefited more from motor training than from cognitive or cognitive-motor dual-task training (H2). Finally, we reasoned that cognitive-motor dual-task performance in persons with higher baseline cognitive and motor fitness benefited more from cognitive-motor dual-task training than from cognitive training alone or motor training alone (H3).  **Example b:** The primary objective of this trial is to assess whether a 12-week strength-training program improves lower-extremity strength in older adults compared with a control (no exercise) group. We hypothesize that participants receiving strength training will show greater gains in lower-extremity strength (measured by a chair-stand test) than those in the no-exercise group. | | |
| **3 Methods** | | | | | | | |  |
| **3 Trial design** | | | | | | | |  |
| 3a | | Describe trial design (such as parallel, cluster, factorial) including allocation ratio | | - Ensures proper reporting of study design, which is crucial to interpret the effect of single or multiple combined interventions. - Relevant to understand potential complex interactions in factorial design for example. Such designs enable evaluations of two or more interventions simultaneously by combining them in the same study. For example, a 2×2 factorial design can include four distinct groups that result from crossing two interventions (e.g., each intervention alone, their combination, control). | | **Example a:** A single-blind, randomized, controlled intervention with healthy older adults is conducted. Three training programs (cognitive, motor, and simultaneous cognitive-motor training) are included. Randomization was conducted at a 1:1:1 ratio using a computer-generated random allocation schedule.  **Example b:** This study employed a parallel, two-arm randomized controlled trial with a 1:1 allocation ratio. Participants were randomly assigned to either a 12-week strength-training program or a no-exercise control group. | |  |
| 3b | | Describe important changes to methods after trial commencement (such as eligibility criteria), with reasons | | - Ensures transparency - Allows accurate risk of bias assessment | | **Example b:** The trial was registered at clinicaltrials.gov as NCT …(other trial register are possible). The secondary outcome ‘trait anxiety’ was added after the preregistration during the implementation of the protocol because it was expected a possible moderating effect of this factor on the effect size of the primary outcome. | |  |
| **4 Participants** | | | | | | | |  |
| 4a | | Eligibility criteria for participants | | - Ensures understanding understand the cohort of interest and recruitment strategies. - Helpful for understanding the mechanism underlying the results obtained. | | Older adults, (N = 128) between 65 and 75 years of age took part in this study. Inclusion criteria comprised: (1) aged between 65 and 75 years (minor exceptions were made for couples for ethical reasons: < 65 and > 75 years), (2) right-handed, (3) active car driving at least once a week within the last 6 months, (4) ability to walk unassisted without self-reported problems (e.g., difficulty to breath, pain, and cardiac palpitations), and (5) community-dwelling. Exclusion criteria comprised: (6) BMI > 30, (7) red-green deficiency or red-green-color blindness, (8) orthopedic impairments, (9) perceived health concerns, (10) neurological diseases, (11) cardiovascular disorders, (12) previous heart attack or stroke, or (13) previous head/brain surgery. In addition, all participants had to obtain a physician’s health clearance (exercise electrocardiogram, ECG) within the last six months. Subsequent screening assessed: (1) overall cognition by the MMSE with a cutoff score of 27/30, (2) visual acuity by the Freiburg vision test (FrACT 3.9.0) with a cutoff score of 20/60, and (4) handedness by the Edinburgh Handedness Inventory. | |  |
| *4a1* | | Including mean age or median of sample age depending on the distribution of your sample, and age range | |  |  |  |  |  |
| *4a2* | | Inclusion/exclusion criteria | |  |  |  |  |  |
| 4b | | Table comparing the main characteristics of the different groups of participants | | - Ensures proper characterization of the study population and support external validity. - Allows assessment of baseline comparability between intervention and control groups. - Helps identify potential confounding factors that may influence intervention effects. - Ensures replication, and generalizability of the study findings. | | 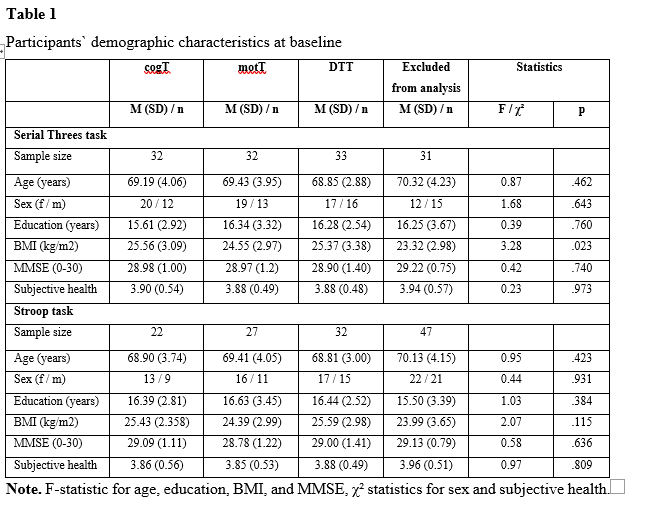 | |  |
| *4b1* | | Level of physical activity or functional capacity at baseline (including method of assessment): level of physical activity in METS.h/week (measured with questionnaires or actimeters), functional capacity (assessed with functional tests such as the 6-min walk test) and exercise capacity (such as VO2max or gait speed). | |  |  |  |  |  |
| *4b2* | | Percentage of women in each group | |  |  |  |  |  |
| *4b3* | | Mean level of education (and SD) of the participants in each group, specify the years or degree | |  |  |  |  |  |
| *4b4* | | Health status: includes the cognitive status of the participants (assessed with MMSE or MoCA, the comorbidities (e.g., depression severity, metabolic disease, diabetes) and pharmacological therapies (e.g., betablockers, antidepressant drugs, sedative drugs). | |  |  |  |  |  |
| *4b5* | | Social support (the help that the participants receive from caregivers.) | |  |  |  |  |  |
| If the study design includes technology support or usage, describe: | | | | | | | |  |
| *4b6* | | Experience with technology | |  | |  | |  |
| *4b7* | | How the participants got access to the technology | |  | |  | |  |
| *4b8* | | Report on digital literacy | |  | |  | |  |
| *4b9* | | Report on any aspects of tailoring the technology | |  | |  | |  |
| 4c | | Settings and locations where the data were collected | |  | | This study was conducted at the University of xx, and at the xx University of Technology, Country. | |  |
| **5 Interventions** | | | | | | | |  |
| 5a | | Describe the interventions for each group with sufficient details (if multimodal for every content or modality) to allow replication, including how and when they were actually administered, including description of: | | - Accounts for the different adaptation induced by each exercise type. Exercise frequency, duration, and intensity can moderate the effect of exercise on several outcomes. | | Three different training programs (cognitive training, motor training, cognitive-motor dual-task training) were conducted at the facilities. They lasted twelve weeks and included two one-hour training sessions per week (24 training sessions in total). Each of the three training programs included a total of 72 15-minute training blocks (18 hours in total). The blocks were performed several times in a predefined sequence that was the same for all participants. In each training session, three of those blocks were provided to the participants. To ensure continuous training progress, the difficulty level of the training was continuously adjusted to individuals’ performance. The three different training programs are briefly described below. For a more detailed description of the training programs and exemplary training sessions, please refer to our study protocol. To ensure that training effects were not confounded by the effects of cardiovascular practice on brain functions, the training intensity of the motor and multitask training did not exceed 60% of VO2-peak. Apart from participating in the training, participants were asked not to alter their regular daily routines, including social, physical, and cognitive activities. See Figure 2 for a photographic illustration of the three training programs. All training was supervised by trained staff.  **Cognitive training:** The training program was conducted in a computer pool with a separate computer per participant. The exercises were presented on a computer monitor and handheld trackball mice (YUMQUA Y-01, YUMQUA, Shenzhen, China) were used to control the cursor. This input device was chosen for compatibility with cognitive-motor training, where conventional computer mice would not be practical (see below). The training program included 22 different cognitive exercises from three different software applications: NeuroNation (NeuroNation, Berlin, Germany), Happyneuron (Scientific Brain Training, Lyon, France), and Neuropeak. The exercises trained different fluid cognitive functions, specifically inhibitory control, updating, shifting, multitasking and action planning which are essential for everyday life functioning. One exercise was performed in each of the 72 training blocks. Throughout the training, the different exercises were performed several times (approximately three times), and exercise difficulty increased adaptively with participants’ proficiency based on algorithms of the software. Group sizes ranged from 10 to 15 participants at Institution xy (provide an institution here).  **Motor training :** The training program was conducted in a customized exercise room and consisted of 15-minute blocks of floor exercises and walking exercises. The floor program included various exercises that train either strength or balance. The difficulty of the exercises was varied with different surfaces (e.g. AIREX-Pad, Balance Board). Various flexibility exercises were performed for recovery between and after the strength and balance exercises. The walking program was performed on a non-motorized treadmill with curved belt (Speedfit SpT-1000C, Tobeone, Korea). It included different walking exercises with varying degrees of difficulty. Throughout the training, the different exercises were performed several times, and exercise difficulty increased adaptively with participants’ proficiency. Group sizes ranged from 10 participants at Institution xy (provide an institution here).  **Cognitive-motor dual-tasking training :** This training was conducted in the same exercise room as motor training. Participants performed the cognitive and motor exercises simultaneously (e.g., they performed a cognitive exercise while standing on one leg). Thereby, the execution and sequence of exercises stayed exactly the same as in the other two training groups. Again, exercise difficulty increased adaptively with participants’ proficiency. The cognitive exercises were presented on a 48″ screen placed at eye level in front of the participant. Group sizes ranged from 10 participants at TUC and one to two participants at UM. | |  |
| *5a1* | | Exercise type (single mode or multimodal) | |  |  |  |  |  |
| *5a2* | | Exercise frequency: the number of sessions per week | |  |  |  |  |  |
| *5a3* | | Duration of exercise: length of time spent on each exercise session in the intervention (in minutes) | |  |  |  |  |  |
| *5a4* | | Exercise intensity: the level of difficulty or effort exerted during the exercise in the intervention (and methods used to assess and monitor it): the percentage of HR max/HRreserve/VO2max/VO2peak; Rating of perceived exertion scale/fatigue before and after intervention /report also the scale (i.e. 6-20, 1-10). | |  |  |  |  |  |
| *5a5* | | Duration of the whole intervention: For chronic exercise interventions, report the total duration of the intervention. For acute exercise interventions: In pre-post designs, report the time interval between the two exercise bouts (in minutes). In multi-session protocols, report the time between sessions (in days). | |  | |  |  |  |
| 5b | | Describe exercise physiology aspects (cardiovascular, metabolic, muscular and other potential adaptations) | | - Ensures replication of the proposed training program and accurate interpretation of the results. - Allows comparison between studies using different modalities: resistance, aerobic exercise, interval training forms (i.e. high-intensity interval training) - Essential for clear reporting of multi-modal exercises where all guidelines need to be reported for the single components, a table is recommended to report the details | | **Example a:** To ensure continuous training progress, the difficulty level of the training was continuously adjusted to individuals’ performance. To ensure that training effects were not confounded by the effects of cardiovascular practice on brain functions, the training intensity of the motor and multitask training did not exceed 60% of VO2-peak.  **Cognitive training :** The exercises were presented on a computer monitor and handheld trackball mice (YUMQUA Y-01, YUMQUA, Shenzhen, China) were used to control the cursor.  The training program included 22 different cognitive exercises from three different software applications: NeuroNation (NeuroNation, Berlin, Germany), Happyneuron (Scientific Brain Training, Lyon, France), and Neuropeak.  **Motor training :** Various flexibility exercises were performed for recovery between and after the strength and balance exercises.  **Cognitive-motor dual-tasking training:** The cognitive exercises were presented on a 48″ screen placed at eye level in front of the participant.  **Example b:** Patients were asked to walk on the treadmill at a precise speed regulated by the exercise physiologists in charge for supervising all the sessions. A fixed walking time of 3 minutes, interspersed by a 1-minute resting pause, to be repeated 8 consecutive times was scheduled. The first two units/ levels of walking were performed at 2,0 km/h; the third and fourth at 2,5 km/h; the fifth and sixth at 3,0 km/h and the last two at 2,5 km/h. Walking speed was increased by 0,1 km/h for all units/ levels throughout all walking sessions. The last of the scheduled 10 training sessions would encompass the following structure (3,0-3,0-3,5-3,5-4,0-4,0-3,5-3,5 km/h). Patients were allowed to use handrails of the treadmill to maintain balance. | |  |
| *5b1* | | Exercise progression: (increase in the difficulty, duration, and frequency of the exercise program) | |  |  |  |  |  |
| *5b2* | | Intensity changes in different exercise types | |  |  |  |  |  |
| *5b3* | | Resting times: duration of rest or recovery between sets or exercises during the intervention | |  |  |  |  |  |
| *5b4* | | Include METs for exercise program/its components: This refers to the estimated energy expenditure during physical activity intervention | |  |  |  |  |  |
| *5b5* | | If technology was used: describe if and how exposure is controlled in the technology | |  |  |  |  |  |
|  | | Describe whether there is any non-exercise component such as diet, mindfulness training, cognitive training, drugs, etc. | | - Essential to understand how each component of the intervention contributes to the effect of the outcome variable | | The control group continued with usual care. Their general practitioner advised them to perform physical activity as recommended by the guidelines. Patients enrolled in this group did not receive any additional exercise program to be performed. | |  |
| 5e | | Describe exercise settings | |  | |  | |  |
| 5e1 | | Whether exercise is performed individually or in a group | | - Informative for discussing the results obtained, including if applicable, all aspects related to technology use. | |  | |  |
| 5e2 | | Whether exercise is supervised or unsupervised and how exercise was monitored | |  |  |  | |  |
| 5e3 | | If technology is used describe how exercise was monitored (e.g., telemonitoring) | |  |  |  | |  |
| 5e4 | | Describe any home program component | |  |  |  | |  |
| 5e5 | | Whether the exercises are tailored or generic (one-size fits all). If tailored, describe how it was tailored to the individual | |  |  |  | |  |
| 5e6 | | Whether there are any simultaneous, consecutive exercise/intervention components (add a short-detailed description.) | |  |  |  | |  |
| 5e7 | | Type of exercise equipment | |  |  |  | |  |
| If technology is used describe: | | | | | | | |  |
| *5e8* | | The design, specific functions (including a list of examples with description), software, interface, adaptation methods, algorithms | |  | | All training was supervised by trained stuff.  **Example a: Cognitive training :**The exercises were presented on a computer monitor and handheld trackball mice (YUMQUA Y-01, YUMQUA, Shenzhen, China) were used to control the cursor.  The training program included 22 different cognitive exercises from three different software applications: NeuroNation (NeuroNation, Berlin, Germany), Happyneuron (Scientific Brain Training, Lyon, France), and Neuropeak  Throughout the training, the different exercises were performed several times (approximately three times), and exercise difficulty increased adaptively with participants’ proficiency based on algorithms of the software. Group sizes ranged from 10 to 15 participants at xx and one to ten at xx.  **Motor training:** Group sizes ranged from 10 participants at TUC and one to two at UM.  **Cognitive-motor dual-tasking training:** The cognitive exercises were presented on a 48″ screen placed at eye level in front of the participant. Group sizes ranged from 10 participants at xx and one to two at xx.  **Example b:** Patients individually performed the walking program at their home in remote connection with the rehabilitation center. Before starting the exercise sessions, participants were asked to wear the tracker device (name, producer…) that was provided to them and click on it to connect with the central hub. During the training sessions, performed according to the instruction form provided to each patient (Table X), the exact duration of the walking and the resting pauses was remotely monitored and recorded. | |  |
| *5e9* | | How progression is programmed | |  |  |  |  |  |
| *5e10* | | How the outcome is calculated if available | |  |  |  |  |  |
| 5e11 | | Specific match between technology of intervention and outcome | |  |  |  |  |  |
| 5f | | Describe type of control group in detail, e.g., was the control group active or passive? If active, what type of activity did they perform? If there is an Active control group, describe it according to 5a1 to 5e5 suggestions | | - Important to interpret the obtained results. | | Participants randomized into the active control group received a fitness tracker and a general oral recommendation to perform at least 15 minutes of continuous walking per day, at least 5 days per week. | |  |
| *5f1* | | Report whether the control group was blinded | |  |  |  |  |  |
| *5f2* | | Describe if and how exposure is controlled for the control group in the technology | |  |  |  |  |  |
| 5g | | Describe motivational control aspects | | - If behavioral change techniques or motivational strategies are employed, this should be described since they may lead to different compliance to the physical activity program. - When applicable, participants’ compensation should be reported since once again it may affect compliance/attendance. | | **Example a:** Adherence to the training sessions was measured by the trainers.  All participants receive monetary compensation (15 € per testing day).  **Example b:** Adherence to the prescribed home-based training sessions was controlled through the diaries that patients received upon randomization. After each exercise session patients were asked to fill the daily log with the total amount of walking (in minutes) and with possible symptoms occurred. The percentage of the adherence to the intervention was calculated upon the completed number on exercise sessions respect to the prescribed ones.  In case of availability, a caregiver was interviewed to double check if the diary was compiled honestly and correctly. In case the diary was not returned, the percentage of exercise sessions completed was directly asked to the patient and to his/her caregiver (blinded to each other answers). If different results occurred, the mean between the two measurements was employed. To reinforce motivation to exercise every two weeks, a member of the research team phone called each patient to give him/her the possibility to ask questions, and to ask if he/she is performing the exercise programs, if symptoms occurred and so on.  According to the Italian laws, participants cannot be compensated when enrolled in a clinical trial. | |  |
| *5g1* | | Describe how compliance/adherence to exercise is measured/assessed | |  |  |  |  |  |
| *5g2* | | Describe of motivational strategies and behavioral change techniques if used | |  |  |  |  |  |
| *5g3* | | If applicable, how do participants get compensated for study participation | |  |  |  |  |  |
| 5h | | Describe extent to which intervention was not delivered as planned if applicable | | - Contributes to the understanding of how unexpected adjustments might have affected outcomes. - Supports transparency, enabling other researchers and practitioners to gauge the feasibility, generalizability, and replicability of the study under real-world constraints. | | Due to COVID restrictions, halfway through the intervention period, the motor and cognitive-motor dual-task training had to be conducted with two participants per group instead of ten. | |  |
| 5i | | Control for confounding factors (e.g. participants’ beliefs about exercise, additional to the intervention physical activity, new treatments started during the study, and preferences for specific interventions) | | - Essential for attributing observed effects to the intervention of interest. By systematically measuring and reporting these factors, researchers can assess how they may influence outcomes, interpret results more accurately, and strengthen the study’s internal validity. | | All people enrolled in either one of the two active exercise group (Aerobic versus Resistance training) were controlled for the starting of treatment with Semaglutide ® throughout all the study duration, for the known possible additional effects of the use of this drug on the primary outcome of the study (e.g. weight loss).  All participants in both groups were otherwise provided with a Fitness tracker (producer, State) to collect data about additional physical activity throughout the entire four weeks of training. | |  |
| *5i1* | | If applicable, report beliefs and stereotypes of participants concerning the effects of regular exercise on health | |  |  |  |  |  |
| *5i2* | | Physical activity (PA) practiced by the participants beside the intervention. The method to measure PA level (cf. 4b1) should be mentioned (e.g., actimeter, questionnaire) | |  |  |  |  |  |
| *5i3* | | If applicable, report participants’ preference for a specific intervention (particularly when several interventions are implemented) | |  |  |  |  |  |
| **6** | | **Outcomes** | | To be consistent with other reporting please refer to CONSORT guidelines for 6 to -8b | | | |  |
| 6a | | Completely defined pre-specified primary and secondary outcome measures, including how and when they were assessed | |  | | System hardware and software: The dual-task walking test was performed with the GRAIL system (Gait Real-time Analysis Interactive Lab, Motekforce Link, Amsterdam, The Netherlands). The GRAIL is a valid and reliable gait assessment device that integrated two embedded force plates in an instrumented 3D split-belt treadmill platform (0.8 × 1.5 m). A semi-cylindrical 240° projection screen (2.4 × 5 m) was located in front of the treadmill. Four RGB projectors connected in series project a virtual scenario onto the projection screen. A photodiode was placed on the projection screen to precisely measure the visual onset of stimuli, thus considering any unsystematic variations in the onset times of the RGB projectors. A custom-made ergonomic key switch in the participants’ dominant hand was used to record manual responses, and a voice recorder was used to assess verbal responses. The system's safety measures included two handrails attached to the side of the treadmill and two laser barriers at the front and rear of the treadmill. Participants also wore a safety harness during walking that was attached to the ceiling to prevent injury in case of a fall. The experimenters had a stop button available to stop the treadmill immediately in case of an emergency. However, no falls or emergency stops occurred. The systems standard software D-Flow (Motekforce Link, Amsterdam, the Netherlands) was used to customize the virtual scenario. It depicted an industrial-like virtual landscape. Motor and cognitive tasks were also designed and integrated within D-Flow. All instructions and tasks were presented at eye level in small rectangular grey and brownish boxes.  Motor and cognitive tasks: Motor and cognitive tasks were presented in a mixed sequence comprising six different tasks, with five trials each. The sequence remained consistent across all participants and for both the pre-test and post-test. No task was repeated consecutively for more than two trials. Each trial lasted 30 seconds and was preceded by an additional 3-second introductory text (for example “Standing only” or “Walking only,” in German). The entire set of tasks spanned 16.5 minutes (30 x 30 seconds + 30 x 3 seconds).  Testing consisted of one baseline task, three tasks in ST condition encompassing one motor and two cognitive tasks, and two combined tasks in DT condition. Outcome measures for the two combined tasks in DT condition were the same as described for the tasks in ST condition. The tasks were as follows: (1) Standing task (baseline): Participants remained stationary, standing with both feet on the treadmill and maintaining a forward gaze directed at a fixation cross. Ground reaction forces were recorded. This task was not analyzed in this study. (2) Walking task (ST condition): Participants walked at a fixed treadmill speed of 1 m/s, focusing their gaze straight ahead on a fixation cross. Since the treadmill accelerated and decelerated at a rate of 0.2 m/s², it necessitated a 5-second transition between standing and walking trials. Ground reaction forces were recorded. (3) Serial Threes task (ST condition): Participants maintained a stationary position on the treadmill while focusing on the fixation cross at the center of the projection screen. A three-digit number was presented at the start of the trial for 5 seconds. Based on this number, participants were required to count backwards in threes from this number, as rapidly and accurately as they could, and to verbalize each resultant number. They had to keep their eyes open throughout the task, to articulate each number in full (for instance, stating “177” rather than “77”), and to refrain from correcting any mistakes. That is, they had to continue counting from the last number stated, even if incorrect. All verbal responses were documented by the experimenter and additionally captured via a voice recorder. (4) Color Word Stroop task (ST condition): This task evaluated inhibitory control by presenting the four color-naming words yellow, red, blue, green in a randomized sequence. Each word appeared for 500 ms, followed by a fixation cross for 1800 to 2200 ms, such that the average inter-stimulus interval (ISI) was 2500 ms. Stimulus words were congruent, i.e., the color of the word matched its meaning (e.g., "green" appeared in green), or incongruent i.e., the color and meaning differed (e.g., "green" appeared in blue). Two response options were shown for 1500 ms, aligned with the onset of the stimulus. These options were displayed in two rectangular areas, one to the left and one to the right below the stimulus word, both in white font. One response indicated the color of the font of the stimulus word, and the other named one of the three other possible colors. Participants had to decide which of the two response words corresponded to the color of the font by pressing either the left or the right button on a handheld key switch. They were instructed to respond as quickly and accurately as possible. The Stroop task design maintained a balance across various factors: 50% of the trials were congruent and 50% were incongruent, each font color was used in 25% of trials, and the positioning of correct and incorrect answers, as well as the frequency of these answers per color, were equally distributed at 50%. Reaction times and accuracy of responses were meticulously recorded via the handheld key switch responses. (5) Walking + Serial Threes task (DT condition): Participants concurrently performed the Walking and Serial Threes tasks, without prioritizing either. (6) Walking + Color Word Stroop task (DT condition): Participants concurrently performed the Walking and Color Word Stroop task, without prioritizing either.  Procedure: Participants familiarized themselves with the treadmill by walking through a simulated forest environment for approximately 5 to 10 minutes, during which the walking speed gradually increased to 1 m/s. The familiarization phase concluded once participants were able to walk steadily, while maintaining their focus on the center of the projection screen. Subsequently, cognitive tests such as the MMSE and DSST were conducted. These lasted about 12 to 15 minutes in total, allowing participants to return to a physical resting state. Following these assessments, participants engaged in a brief practice session lasting about 2 minutes, which included a shortened version of each task in a predetermined sequence. | |  |
| 6b | | Any changes to trial outcomes after the trial commenced, with reasons | |  | |  |  |  |
| **7 Sample size** | | | | | | | |  |
| 7a | | How sample size was determined | |  | | A power analysis was conducted before data collection and reported in detail in the study protocol. The estimated required sample size to provide sufficient power to detect a small to moderate effect was N = 118. With our final sample of N = 97 participants for the Serial Threes task and N = 81 participants for the Stroop task, we did not reach this calculated sample size, due to higher drop-outs and missing data than expected. However, since we conducted our analysis using linear mixed models (LMMs) we are optimistic this will mitigate the shortfall. LMMs are advantageous because they leverage the repeated measures within subjects using disaggregated data, effectively increasing statistical power and requiring fewer participants compared to traditional mean-based statistical analyses on aggregated data. Additionally, it is possible to further increase statistical power by reducing model complexity and selecting the most parsimonious model, while also effectively balancing type I and type II error. | |  |
| 7b | | When applicable, explanation of any interim analyses and stopping guidelines | |  |  |  |  |  |
| **8 Randomization,** Sequence generation | | | | | | | |  |
| 8a | | Method used to generate the random allocation sequence | |  | | Randomization was conducted at a 1:1:1 ratio using a computer-generated random allocation schedule. | |  |
| 8b | | Type of randomization; details of any restriction (such as blocking and block size) | |  | |  | |  |
| 8c | | Use of a minimization process. Example: randomization according to baseline physical and cognitive levels of the participants | | - Especially in RCTs dealing with a rare condition, when a low number of participants should be enrolled, minimization, that is an accepted randomization method (Altman, 1991), may be applied. | | Consider a study that aims to evaluate a 12-week strength-training program in older adults who have varying cognitive levels (assessed by a standard cognitive screening tool i.e. MMSE). Researchers assign the first participant randomly. For each subsequent participant, they look at cognitive test scores and age, then allocate that participant to whichever group would best maintain balance in those variables. If both arms are already balanced, they might randomize the next individual with a higher probability of going to the group that would help keep the balance intact. | |  |
| **9 Allocation concealment mechanism** | | | | | | | |  |
|  | | Mechanism used to implement the random allocation sequence (such as sequentially numbered containers), describing any steps taken to conceal the sequence until interventions were assigned | | - Ensures proper allocation concealment prevents selection bias and maintains the integrity of the randomization process. Concealing the allocation sequence (e.g., sealed envelopes, software-based allocation) safeguards against bias when enrolling and assigning participants to groups. | | **Example a**: A research assistant sealed the random assignments in envelopes, which were given to participants after completing the pre-tests.  **Example b:** The researchers prepared 100 sequentially numbered, opaque, sealed envelopes containing group assignments for a 2-arm strength-training trial in older adults. Each envelope was opened only after the participant has given informed consent and completed baseline measures, thus ensuring the allocation sequence remains concealed until interventions were assigned. | |  |
| **10 Implementation** | | | | | | | |  |
|  | | Who generated the random allocation sequence, who enrolled participants, and who assigned participants to interventions | | - Increases transparency, reduces risk of bias, and ensures that responsibilities are appropriately allocated | | The random allocation sequence was generated by a researcher working in the project. A research assistant sealed the random assignments in envelopes, which were given to participants after completing the pre-tests. | |  |
| **11 Masking** | | | | | | | |  |
| 11a | | Was a masking or blinding applied, and if yes, who was blinded after assignment to interventions (e.g., participants, care providers, those assessing outcomes), and how | | - Important for assessing potential bias and ensuring that outcomes are measured objectively. Reporting any changes to blinding procedures provides clarity about the study’s execution and potential influences on the results. | | **Example a:** Outcome assessors were blinded to group allocation, and personnel delivering the intervention were blinded to outcome assessments and individual performances. To minimize experimental contamination from social interaction and communication among participants, they were instructed not to discuss their assigned interventions with each other. Close friends and spouses were assigned to the same intervention groups.  **Example b:**  Both participants and outcome assessors were blinded to group allocation when feasible (e.g., by keeping assessment staff unaware of which intervention participants received). If, during the study, the blinding of outcome assessors was unavoidably compromised (e.g., an assessor discovered a participant’s group through conversation), the incident was documented in a protocol amendment, along with a rationale and an explanation of the potential impact on the outcomes. | |  |
| *11a1* | | If there were any blinding changes, report it | |  |  |  |  |  |
| 11b | | If relevant, describe similarity of interventions | | - Ensures that comparable activities, settings, equipment, or session lengths are used helps to isolate the specific variable of interest (e.g., exercise intensity) and reduces confounding factors. Similarity also minimizes the influence of extraneous differences (e.g., environment, schedule) on study outcomes. | | **Example a**: Three different training programs (cognitive training, motor training, cognitive-motor dual-task training) were conducted at TUC and MU facilities. They lasted twelve weeks and included two one-hour training sessions per week (24 training sessions in total). Each of the three training programs included a total of 72 15-minute training blocks (18 hours in total). The blocks were performed several times in a predefined sequence that was the same for all participants. In each training session, three of those blocks were provided to the participants. To ensure continuous training progress, the difficulty level of the training was continuously adjusted to individuals’ performance.  **Example b:** Both groups trained three times per week in the same nursing home facility, with equal session durations (45 minutes). | |  |
| **12 Statistical methods** | | | | | | | |  |
| 12a | | Statistical methods used to compare groups for primary and secondary outcomes | | - Enhances reproducibility and transparency. - Allows readers to assess the appropriateness of the chosen analytical approach and facilitates comparisons across similar studies. | | **Example a:** For all analyses, we applied LMMs using the lme4 package. All models were fitted using maximum likelihood estimation (ML), which is assumed to provide better estimates for fixed effects than restricted maximum likelihood estimation (REML). We further followed the parsimonious model selection procedure proposed by Bates et al., which is described in more detail in the supplementary material.  All models were built separately for the combination of walking and Serial Threes (S3), and for the combination of walking and Stroop (STR), administered as pre- and post-tests. This was done across both the ST and DT conditions, with performance (cognitive performance and motor performance) as the dependent variable. The following independent variables were included in the models: Time (pre-test [pre] vs. post-test [post]), condition (single-task [ST] vs. dual-task [DT]), domain (cognitive performance [cog] vs. motor performance [gait]), training group (cognitive training [cogT], motor training [motT], cognitive-motor dual-task training [DTT]), baseline cognitive fitness (cf Ref), and baseline motor fitness (mf). Contrasts were specified with the MASS package [ref ] and the hypr package. Time and condition were dummy coded with pre-test and ST condition as the reference level, allowing these categories to serve as the baseline for comparison. Domain was sum (or effect) coded by cog as negative and gait as positive, with zero as the mean of the two levels. This approach simplifies interpretation of main effects and interactions by balancing the levels [ref]. For group, we specified three contrasts comparing (1) DTT vs. cogT, (2) DTT vs. motT, and (3) cogT vs. motT.  In a first step, we tested for the differential effects of the three interventions (cogT, motT, DTT) on cognitive-motor performance during both ST and DT conditions. Following a pre-specified model selection process (for details see supplements), the final models for this analysis included fixed effects for time (pre, post), group (motT, cogT, DTT), domain (cog, gait), condition (ST, DT), the interaction between condition x domain and the triple-interaction between time x group x domain. The random effects term included time, domain and condition which were allowed to vary within participants. Including factors as both fixed and random effects is a widely accepted and recommended approach in mixed-effects modeling, as it allows for the estimation of population-level effects while accounting for within-subject variability [ref] However, to avoid overfitting and ensure model stability, we evaluated the random effects structure by checking model fit using the diagnostic functions isSingular and rePCA as part of the lm4 package. The final model was as follows: cognitive and motor performance ~ 1 + time * group * domain + condition + condition : domain + (1 + time + domain + condition \| Subj), in the model formula, * denotes all main effects and interactions between the variables, : specifies only the interaction between two variables without their main effects. Because we were not only interested in the time x group x domain interaction, but also in the time x group interaction within the separate domains (cog vs. gait), we rebuilt the final models to have these effects nested within the levels of the variable domain [ref]: cognitive and motor performance ~ 1 + domain / (time * group) + condition + condition : domain + (1 + time + domain + condition \| Subj).  In a second step, we evaluated the influence of baseline cognitive and motor fitness on the intervention effects using model comparisons with log-likelihood ratio tests. Specifically, we constructed a model similar to the one detailed above. However, the fixed effect of the three-way interaction of time x group x domain was replaced with the fixed effects of two four-way interactions of time x group x domain x cf and time x group x domain x mf. This new model (cognitive and motor performance ~ 1 + time * group * domain * (cf + mf) + condition + condition : domain + (1 + time + domain + condition \| Subj)) was then compared to the original model to test whether cognitive and motor fitness influence the outcomes. If model comparison showed significance, it was further compared to the following models: 1) a model including only the four-way interaction of time x group x domain x cf, to test the moderation effect of cf alone, and 2) a model including only the four-way interaction of time x group x domain x mf, to test the moderation effect of mf alone. Additionally, 3) a model including a five-way interaction of time x group x domain x cf x mf was compared to a model with the two four-way interactions to test the moderation effect of the interaction between baseline cognitive and motor fitness  Because we were not only interested in the time x group x domain x cf interaction, but also in the (1) time x group x cf interaction within the separate domains (cog vs. gait), and the (2) time x domain x cf interaction within the separate training groups (motT vs. cogT vs. DTT), we rebuilt the final models to have these effects nested within the levels of the variable domain: (1) cognitive and motor performance ~ 1 + domain / (time * group * cf) + condition + condition : domain + (1 + time + domain + condition \| Subj); (2) cognitive-motor performance ~ 1 + group / (time * domain * cf) + condition + condition : domain + (1 + time + domain + condition \| Subj);  For all LMM analyses, t-values above t = 2.00 were considered significant [83,84]. Only significant results are reported with statistical values in the text of the results section, while non-significant results are provided in the tables.  **Example b:** Repeated measures mixed ANOVA (group x time) was used to analyse the results. Tukey’s post-hoc tests were used to determine pairwise differences wherever repeated measures ANOVA showed statistically significant interaction. Statistical significance was set at p < 0.05, and all results were reported as mean ± standard deviation. Pearson’s correlation coefficient was used to assess the relationship between the examined variables. The normality of the distribution was assessed using the Shapiro-Wilk test | |  |
| 12b | | Methods for additional analyses, such as subgroup analyses and adjusted analyses | | - Clarifies how the data were further examined and which participant characteristics might influence the main findings. This increased level of detail fosters transparency and supports readers in evaluating the robustness and potential generalizability of the results. | | Repeated measures mixed ANOVA (group x time) was used to analyse the results. Tukey’s post-hoc tests were used to determine pairwise differences wherever repeated measures ANOVA showed statistically significant interaction. Statistical significance was set at p < 0.05, and all results were reported as mean ± standard deviation. Pearson’s correlation coefficient was used to assess the relationship between the examined variables. The normality of the distribution was assessed using the Shapiro-Wilk test | |  |
| 12c | | Type of analysis used: intention-to-treat, complete-case, per-protocol | | - Evidence-based medicine guidelines recommend to use intention-to-treat analysis because it considers participants who dropped out of the intervention. This type of analysis aims to be as closer as possible to real-world conditions, and then assesses treatment effectiveness while considering factors like protocol adherence. When, a researcher wants to conduct a per-protocol analysis because he/she wants to test a mechanistic hypothesis that necessitates that participants fully adhered to the treatment, it is recommended to conduct both the intention-to-treat and the per-protocol analysis. Complete-case analysis is often used when there were no dropouts or a small number of dropouts during the intervention. | | **Example a:** Data were analysed according to the per-protocol principle.  **Example b:** Data were analysed according to the intention-to-treat (ITT) principle. To minimize type I errors and loss of power, endpoint analyses according to the imputation of the mean of the other group principle were performed for missing values at six months (ref) | |  |
| *12c1* | | If the intention-to-treat analysis was used, which imputation technique was carried out to replace missing data, if per-protocol analysis, what protocol was not followed | | - Favor intention-to-treat analysis as much as possible. For a presentation of differences and similarities, advantages and disadvantages between intention-to-treat and per-protocol analyses (Molero-Calafella et al., 2024) The imputation technique used by the authors needs to be specified to allow the reader appreciates the impact of the approach used on the results. | |  | |  |
| **13 Results** | | | | |  | | |  |
| 13a | | For each group, the numbers of participants who were randomly assigned, received intended treatment, and were analyzed for the primary outcome | | | - Illustrates participant flow, attrition, and adherence. - Helps readers evaluate whether differences in group sizes or dropout rates could have influenced the results and ensures an understanding of how participants progressed through the study. | | **Example a:** Data from 97 participants were analyzed for the Serial Threes task, and data from 81 participants for the Stroop task.  **Example b:** In the exercise group, 40 participants were randomly assigned, 35 received the intended program, and 30 completed final assessments for muscle mass. In the control group, 40 were assigned, 38 received no structured exercise, and 32 were analysed for muscle mass. |  |
| 13b | | For each group, losses and exclusions after randomization, together with reasons | | |  |  | Exclusion from the data analysis was due to missing data from participants dropping out because of illness or time constraints, as well as data collection issues caused by technical problems or participants having difficulties performing the tasks. |  |
| 13c | | For each group, percentage of compliance/adherence to the intervention | | | - Crucial for understanding the feasibility and safety of the intervention. - Offers insights into how effectively participants engaged with the study protocol and whether extraneous factors influenced outcomes. - Increases transparency and helps contextualize study findings, particularly when additional technology-based tools are used | | **Example a**: Except for two participants, who completed 21 and 22 sessions respectively, all participants completed the planned 24 sessions.  **Example b:** A total of 64 home-based walking sessions were scheduled for the total duration of the program.  Basing on the data collected through the fitness tracker, 50% of participants (n=10) completed the 100% of prescribed sessions; 25% of them (n=5) completed between 75% and 99% of the session, while the remaining 5% of patients (n=5) completed less than 50% of the training sessions.  No falls, symptoms or adverse events related to the exercise program occurred during the study period. One patient referred to the local hospital for an eye problem not related to the exercise performed. |  |
| *13c1* | | Describe if adverse events like injuries occur, number, dropouts, and reasons for dropouts, this also has to be reported if technology was used | | |  |  |  |  |
| **14** | | **Recruitment** | | | | | |  |
| 14a | | Dates defining the periods of recruitment and follow-up | | |  | | Assessments were conducted at xx between March 2019 and December 2019, and at xx between September 2020 and August 2021. |  |
| 14b | | Why the trial ended or was stopped | | |  | | The trial concluded as scheduled, based on the predefined timeline and available staff resources. |  |
| **15** | | **Baseline data** | | |  | |  |  |
| 15a | | A table showing baseline demographic and clinical characteristics for each group as well as significant differences between the groups (cf. 6) | | | Because the older population is highly heterogenous, the age and a measure of uncertainty (preferably the range of actually recruited participants) should be reported to allow interpretation, reproducibility and generalizability of the results. | | At baseline, no significant differences were observed in age for the two groups: (Exercise: 75±8 years versus Control: 74±9 years; p=0.88). |  |
| *15a1* | | Mean age or median and age range per group need to be reported | | |  |  |  |  |
| **16** | | **Numbers analyzed** | | | | | |  |
|  | | For each group, number of participants (denominator) included in each analysis and whether the analysis was by originally assigned groups | | |  | | Data from 97 participants were analyzed for the Serial Threes task, and data from 81 participants for the Stroop task. |  |
| **17** | | **Outcomes and estimation** | | |  | |  |  |
| 17a | | For each primary and secondary outcome, results for each group, and the estimated effect size and its precision (such as 95% confidence interval) | | |  | | Moreover, for the Serial Threes task, the results demonstrated no significant time x group interaction, but a significant time x group x domain interaction: pre-post changes varied for S3cog and S3gait for DTT vs. motT (Est = -0.28, SE = 0.10, t = -2.69) and motT vs. cogT (Est = -0.24, SE = 0.11, t = -2.25), but not for DTT vs. cogT.  Moreover, results demonstrated significant time x group x domain x cf interactions: for DTT vs. motT (Est = 0.01, SE = 0.00, t = 3.64) and motT vs. cogT (Est = 0.01, SE = 0.00, t = 5.35), but not for DTT vs. cogT, pre-post changes of the two domains were moderated differently by cf (Table 4). |  |
| 17b | | For binary outcomes, presentation of both absolute and relative effect sizes is recommended | | |  | |  |  |
| **18** | | **Ancillary analyses** | | | | | |  |
|  | | Results of any other analyses performed, including subgroup analyses and adjusted analyses, distinguishing pre-specified from exploratory | | |  | | For the Serial Threes task, results showed a significant domain x condition interaction (Est = 0.41, SE = 0.04, t = 10.10; Table 2). While S3cog substantially decreased from ST to DT conditions, S3gait increased slightly from ST to DT. |  |
| **19** | | **Harms** | | | | | |  |
| 19a | | All important harms or unintended effects in each group (for specific guidance see CONSORT for harms) | | |  | | Three of 15 patients in the exercise group dropped out from the study since they reported difficulties in using the smartphone application that was provided to them. Their complaints are reported in Table X. |  |
| *19a1* | | Describe if adverse events like injuries occurred, number of dropouts, and reasons for dropouts | | |  |  |  |  |
| *19a2* | | If technology was used: report on adverse events like injuries, number and reasons for dropouts according to the technology | | |  |  |  |  |

|  | **Discussion** |  |  |
| --- | --- | --- | --- |
| **20** | **Limitations** |  |  |
| 20a | Trial limitations, addressing sources of potential bias (e.g., heterogeneity of characteristics, differences in baseline characteristics, changes in blinding), imprecision, and, if relevant, multiplicity of analyses; | - Provides a clear description very useful for addressing the GRADE items in case of a meta-analyses for having a summary of findings. | This RCT has several strengths but also some limitations. One key strength is that, by including motor, cognitive, and dual-task training in our intervention design, we were the first to directly compare the effects of these three training programs on cognitive and motor dual-task performance. Earlier trials only compared two of these training approaches. Additionally, by structuring the dual-task training to include exactly the same exercises as those used in the motor and cognitive training groups, we eliminated the possibility that the effects of dual-task training were due to different types of exercises rather than the dual-task nature of the training itself. However, our design and setup also come with some limitations. First, we cannot draw conclusions on how the effects would compare to an active or passive control group, such as stretching or waitlist control. However, this was not our primary goal, as there is already substantial literature on this topic. Instead, we aimed to examine the differential effects of cognitive, motor, and cognitive-motor dual-task training, as well as the moderating role of baseline cognitive and motor fitness. Second, during dual-task training, participants received direct feedback on their performance for the cognitive exercises, with progress visualized on a colorful screen. In contrast, instructions for the motor exercises were verbally communicated by the trainers. This difference may have made it more challenging for some participants to focus equally on both motor and cognitive training, potentially leading them to prioritize the cognitive tasks. |
| 20b | Unexpected changes regarding the whole study protocol |  |  |
| 20c | Whether and how the results answer the RQ |  |  |
| 20d | Discuss imprecision, relevant analyses differences in changes in different groups |  |  |
| 20e | If surrogate markers answer RQ |  |  |
| 20e | All aspects of 5i |  |  |
| **21** | **Generalizability** | | |
|  | Generalizability (external validity, applicability) of the trial findings | - Important to elaborate on the difference between significant results and the effect sizes to gain insights into the clinical relevance or meaning of the results. | **Example a:** Due to divergent findings between the two cognitive tasks—Serial Threes and Stroop—no definitive conclusions can be drawn regarding the generalizability of the moderation effects of cognitive and motor fitness on the intervention outcomes.  **Example b:** Three of 15 patients in the exercise group dropped out from the study since they reported difficulties in using the smartphone application that was provided to them. Their complaints are reported in Table X. |
| 21a | Report and discuss the effect sizes |  |  |
| **22** | **Interpretation** | | |
|  | Interpretation consistent with results, balancing benefits and harms, and considering other relevant evidence |  | Our findings suggest that baseline motor fitness, as well as the interaction between baseline cognitive and motor fitness, did not impact the intervention effects for either task. In contrast, baseline cognitive fitness was found to significantly moderate the intervention effects for the Serial Threes task. This differential impact of baseline cognitive and motor abilities on training outcomes aligns with and extends prior research. The moderation effect of cognitive fitness is consistent with the findings of Strobach and colleagues, who demonstrated that reaction time variability (as a proxy for inefficient neural processing) moderates the intervention effects of cognitive dual-task training on cognitive dual-task performance in both young and older adults. The absence of a moderating effect of motor fitness further supports the notion that dual-task walking performance is more strongly linked to cognitive, rather than motor capabilities in older adults. |
| 22a | Discuss all possible confounders that might change the demonstration effect | - Important to report any confounding factors, whether intentional or unintentional on the part of the researchers, that may have a direct or indirect impact on the outcomes observed in the study. | **Example a:** The moderation of cognitive fitness for the Serial Threes task, but not for the Stroop task, may be explained by specific key features of the tasks. One potential explanation is that the self-paced mode of the Serial Threes task allows participants to apply personalized strategies learned during training to optimize their performance (e.g., counting backwards in sync with their walking rhythm). In contrast, the fixed-paced Stroop task imposes strict temporal constraints, limiting opportunities for strategy deployment and reducing the moderating influence of cognitive fitness. Another explanation is that the Serial Threes task relies heavily on verbal responses, whereas the Stroop task depends on manual motor responses. These manual motor responses primarily rely on simpler sensorimotor pathways, making the Stroop task less directly associated with cognitive fitness compared to the Serial Threes task.  **Example b:** The lack of statistically significant differences may be attributed to the fact that patients randomized to the Control group during dialysis sessions were seated next to patients enrolled in the Exercise group. The latter explained the home-based exercise program they had been prescribed and told their control group counterparts that they felt better, had greater independence, and increased strength. Subsequently, members of the control group asked to see the documentation provided to the exercise group patients and began following the same program. This was confirmed by the control group patients themselves to the examiner who conducted the outcome measurement session at the end of the study. |
|  | **Open Science** | | |
| **23** | **Registration** | | |
|  | Registration number and name of trial registry |  | This trial was (retrospectively) registered at xx Register (No xy). |
| **24** | **Protocol** | | |
|  | Where the full trial protocol and the statistical plan can be accessed, if available | Please refer to CONSORT guidelines | A study protocol has been published |
| **25** | **Data sharing** | | |
|  | Where and how the data, statistical code and any other materials can be accessed | Please refer to CONSORT guidelines | The data used during the current study are available at [web link to dataset] |
| **26** | **Funding** | | |
|  | Sources of funding and other support (such as supply of drugs), role of funders, support by training/software development companies | Please refer to CONSORT guidelines | The study was funded by the xxResearch Foundation (xx) and is part of the xx Program (grant No.). The funding body doesn’t play any role in the design of the study, the collection, analysis and interpretation of data and the decision to write and publish manuscripts. The study protocol has not been peer reviewed by the funding body. |

Consolidated Standards of Reporting Trials (CONSORT), Population, Intervention, Comparison, Outcome (PICO), Mini Mental State Examination (MMSE), Montreal Cognitive Assessment (MoCA),

Altman DG. Randomisation. BMJ 1991;302:1481-2. 23  Pocock SJ. Clinical trials: a practical approach. John Wiley, 1983     CONSORT 2010 Explanation and Elaboration: updated guidelines for reporting parallel group randomised trials. BMJ 2010; .doi: <https://doi.org/10.1136/bmj.c869>

Molero-Calafell, Javier, et al. "Intention to treat and per protocol analyses: differences and similarities." Journal of Clinical Epidemiology 173 (2024): 111457.
